# Understanding the contexts and mechanisms that drive high-quality maternity care outcomes within organisations: generating 11 initial programme theories as phase 1 of a realist review

**DOI:** 10.1136/bmjopen-2026-119351

**Published:** 2026-08-31

**Authors:** Claire Feeley, Ellen Thaels, Andrew Booth, Michelle Quashie, Farrah Pradhan, Kate Greenstock, Alicia Burnett, Kate Walker, Yan-Shing Chang, Gordon Prescott, Katrina Rigby, Soo Downe

**Affiliations:** 1 Florence Nightingale Faculty of Nursing, Midwifery & Palliative Care, King’s College London, London, UK; 2 SCHARR, The University of Sheffield, Sheffield, UK; 3 King’s College London, London, UK; 4 Royal College of Obstetricians and Gynaecologists, London, UK; 5 City University, London, UK; 6 University of Nottingham, Nottingham, UK; 7 Lancashire Clinical Trials Unit, University of Lancashire, Preston, UK; 8 Lancashire Teaching Hospitals NHS Foundation Trust, Preston, UK; 9 University of Lancashire, Preston, UK

**Keywords:** Safety, Maternal medicine, Systematic Review, Quality in health care

## Abstract

**Abstract:**

**Objectives:**

There has been considerable attention placed on ‘failures’ of maternity care in high-income countries, with little focus on the drivers of ‘high-quality’ care delivered at the organisational level. We conducted a realist review with this central focus and report on the first phase, initial programme theories (IPTs) generated from the literature, where a future phase will ‘test’ the IPTs.

**Design:**

A realist review methodology was employed, an interpretative, theory-driven approach designed to explore ‘what works, for whom, in what circumstances’ using context, mechanism and outcome (CMO) propositions.

**Data sources:**

The search strategy used a purposive database (MEDLINE) and hand searches, including peer-reviewed journals, grey literature and policy reports, to identify literature rich and relevant to the research question.

**Eligibility criteria for selecting studies:**

As per realist review approaches, we included any English-language literature from peer-reviewed journals, grey literature, policy reports, service guidance and public or professional commentary, from high-income countries, that provided explanatory insights to the operationalisation of ‘high-quality’ maternity care at the organisational level. Using an a priori definition, high-quality maternity care was defined based on four outcome criteria: higher service user and staff well-being, lower prevalence of complications/adverse outcomes and optimal use of resources.

**Data extraction and synthesis:**

Data extraction configured evidence using the ‘If, Then, Leading to’ CMO heuristic. The IPTs were iteratively developed from the synthesised data and refined through consultation with a diverse 26-member core stakeholder group.

**Results:**

The iterative search process included 17 index papers, which were a mix of systematic reviews, primary research studies, commissioned reports, evaluations and commentaries. These papers generated 292 initial CMO hypotheses, which were refined into 11 IPTs focusing on organisational-level mechanisms. The IPTs reflect a ‘both-and’ philosophy, balancing clinical excellence (safety) and values-based relational care (personalisation and staff environment). Key themes include visible and supportive leadership, responsiveness to workload assessments, ensuring operational fitness (system and workplace environment support) and broadening the definition of safety beyond physical safety to include psychological, emotional and cultural safety. The IPT related to ‘A culture of equitable care’ was identified as critical in underpinning all maternity service provision.

**Conclusion:**

The 11 IPTs offer crucial insights into the context and mechanisms that may produce desired outcomes in high-quality maternity care. Learning from high-quality services (safety II) provides a novel approach compared with focusing only on failings. These globally relevant IPTs can be applied to high-income contexts as a foundation for designing future improvement programmes.

**PROSPERO registration number:**

CRD420251104996.

**STRENGTHS AND LIMITATIONS OF THIS STUDY**

- The realist approach is suited to the study of complex adaptive systems such as maternity care.
- The realist approach seeks to provide explanatory insights with real-world applicability.
- The realist approach is methodologically complex, making it challenging to conduct, interpret and communicate effectively.
- As with all reviews, there is a risk of over- or under-interpreting the data, which was mitigated through stakeholder engagement.
- This review focused on high-income country contexts; therefore it may not apply to low- and middle-income countries.

## Introduction

 Globally, there is increasing recognition that the push to centralise labour and birth in health facilities can only result in improved safety *if* the care in those settings is optimal.^1 2^ However, growing concerns target the quality of maternity care in facilities and hospitals around the world.^1–3^ Though most reported concerns originate from the situation in low- and middle-income countries (LMICs),^3^ many also extend to high-income countries. For example, persistent failings in English maternity services have been highlighted by several public inquiries,^4–8^ with a wide-scale national investigation launched in July 2025.^9^ In the English case, the reviews have generated >700 national practice recommendations and changes based on specific cases of error or adverse events.^4–8^ However, simply identifying the problem, while necessary, is not sufficient as the numerous recommendations have not led to the national improvements intended.^10^ Furthermore, Dash *et al*,^11^ recently published a review of patient safety across the health and care landscape in the UK, raising several concerns. They noted that the vast number of agencies tasked with ‘patient safety’ (often narrowly defined) have generated an underwhelming return on investment. Additionally, they found that the generation of a multitude of complex recommendations from so many different agencies makes implementation problematic which has been compounded when recommendations conflict with each other.^11^

While the most recent UK maternity investigation of ^12^ NHS Trusts aims to streamline their review findings into a single set of national actions,^9^ further concerns relate to the inclusion of some of the worst-performing Trusts in the mix to make national recommendations. This approach mirrors ‘Safety I’ thinking, which is based on finding out what goes wrong in a specific case and attempting to rule out this ever happening again across a whole health and social care context.^12 13^ While a Safety I approach might work to control risks that are truly widespread and endemic, generalising to all cases from one specific case of error risks destabilising what currently works, leading to unintended adverse consequences.^12 13^ For example, the Shrewsbury and Telford review^5^ recommended a national review and suspension of the Midwifery Continuity of Care (MCoC) model until all Trusts demonstrated that staffing meets safe minimum requirements on all shifts. However, MCoC was not implicated in this Trust’s failings, and this blanket approach has caused widespread disbanding of effective and functioning MCoC teams.^14 15^ Given the robust evidence in favour of MCoC models and international drive towards whole-scale global implementation^16–19^ this has raised concerns that this recommendation has overall worsened maternity care in England.^20^

In this context, it is prudent to explore alternatives to the Safety I approach, particularly for complex systems such as maternity care. An alternative is to make recommendations based on success, as well as failure—known as a Safety II approach.^13^ A Safety II focus recognises that care goes well most of the time and that, to understand why things may go wrong, both success and failure must be considered together.^13^ Safety II is a new and emerging field not yet widely applied within maternity services. This is a potentially very fruitful area to explore, as less effective maternity units often coexist with higher performing ones.^21^ Acknowledging this context, in our research, we sought to locate and synthesise the current evidence on underlying mechanisms that drive high-quality maternity care around the world. We used a realist review approach^22^ informed by Safety II principles.^13^

Our study builds on existing work located through an initial rapid scoping review, including the seven characteristics of high-performing maternity units described by Liberati *et al*.^23 24^ These characteristics were developed from ethnographic studies of an English hospital with ‘low adverse outcomes’ (not defined in the paper), and four other sites that engaged with a specific collegiate approach to emergency obstetrics training (PROMPT).^24^ They included staff views, but not the views of women and service users, and the characteristics have not been tested for discriminatory power or transferability. Building on this work, and in line with the National Health Service England (NHSE) Better Births policy^25 26^ and recently updated Three Year Delivery Plan,^27^ our study sought to integrate both safety and personalisation, including outcomes of importance to service users, staff and NHS organisations. This work is also aligned with NHSE’s commitment to improving the experiences of care, for both service users and staff.^27 28^ While focusing on English national policy as the rationale for the review, we have included UK and international literature (from similar high-income country contexts) so the findings presented in this paper are relevant to all four UK countries and to wider international maternity care provision where there are sufficient resources to support best practice.

Our a priori definition of a high-performing maternity unit is that it performs better than similarly sized units with similar case mix and demographics according to the MBRRACE-UK criteria^29^ against the following four outcome domains:

- Higher levels of well-being reported by service users.
- Lower prevalence of complications/adverse clinical/psychological outcomes for service users.
- Higher levels of well-being reported by staff.
- Optimal use of resources through judicious use of procedures.

Therefore, the purpose of this paper is to report the first phase of our realist review in which we, together with key stakeholders, developed 11 ‘initial programme theories’ (IPTs) derived from the evidence of what works in maternity care to produce high-performing maternity units. These programme theories can be tested and used in similar high-income maternity contexts around the world as the basis for designing future improvement programmes.

### Rationale for a realist approach and focus of review

Health and social care providers operate with complex systems. This is as true of maternity care as for any other area of health and social care. The socio-ecological model^30^ has been used to capture how each level of the health system is interdependent with the others, focusing on personal, interpersonal, organisational, community and public policy levels. However, studies of good quality care rarely pay attention to factors beyond the personal activities and interactions of staff at the first and sometimes second level of the system in which staff operate. Very good evidence exists on some of the activities and procedures that can improve mortality and morbidity, including social support,^31–33^ midwifery models of care across the continuum, where midwives are embedded within the workforce and health services infrastructures with timely access to medical support if required^16 19 31–35^ and addressing health inequities.^1 16 19 31–38^ There is also growing evidence of the individual drivers for staff well-being, including having the time and support to do the job staff want to do,^39 40^ the importance of psychological safety and effective teamwork^41 42^—all of which are factors in delivering effective high-quality maternity care. We also have some tools for ensuring that the right care is provided by the right staff at the right time to the right service user, for example, the Robson criteria.^41–43^ However, less is known about how organisations deliver services to maximise these beneficial interventions to produce high-quality maternity care.

To address this gap, we decided to use a realist review methodology. A realist review is an interpretative, theory-driven approach designed to explore ‘what works, for whom, in what circumstances’.^22 44^ Attention is placed on ‘contexts, mechanisms and outcomes (CMO)’ that mediate what, why and how something works (or does not).^22 44^ We adopted this approach because while we broadly know ‘what works’ in terms of high-quality maternity care, less is known about how such care is operationalised within complex maternity organisations. Our protocol was published on PROSPERO CRD420251104996 and subsequently we have followed the RAMESES reporting process^45^ (see checklist).

### Aims, objectives and research question

The overall aim of this review is to understand what mechanisms drive high-quality maternity care within organisations to produce positive outcomes across our a priori four domains and in what circumstances these mechanisms occur. Typically, a realist review involves two phases, developing the IPTs and then ‘testing’ and refining those theories either via further literature searching (realist review) or via primary data collection (realist evaluation).^44^ In either case, the development of IPTs constitutes a core step in the realist approach.^46^ Therefore, this paper reports on the first phase, in which 11 IPTs were developed from an extensive international evidence base together with stakeholder input.

#### Research question

The research question for this first phase was ‘What mechanisms drive the successful delivery of high-quality maternity care within organisations?’*.*

#### Objectives for this stage of the review

- To establish a PPI, professional, governance and organisational stakeholder group and a multi-disciplinary steering committee.
- To investigate high-quality maternity care against the four a priori evidence-informed domains (outlined earlier).
- To develop realist IPTs that explain the mechanisms of high-quality maternity as delivered by organisations through an evidence review and stakeholder input.

### Research team, stakeholder engagement and governance

The core research team comprises a multidisciplinary group which includes two midwife-academics as co-leads of the project (CF/SD), two PPI-leads (MQ with an extensive background in maternity advocacy and co-production projects and ABu with an extensive background in the black maternal activist space), an obstetrician (KW), a statistician (GP), a clinical research delivery lead (KR), a Safety II expert (FP), a realist methodologist expert (ABo), a midwife academic specialised in ‘quality’ maternity care (ET), an infant feeding and child health researcher (Y-SC) and a midwife master’s student focusing on delivering ‘great’ maternity care from the perspective of staff (KG).

A key component of realist approaches is the inclusion of stakeholders to explore and refine the research throughout the project.^47 48^ Therefore, following a successful launch webinar, we invited expressions of interest to participate in the core stakeholder group (CSG). From 117 expressions of interest, we sought to maximise the diversity of the group and included 26 diverse stakeholders:

- Four service users (with diverse maternity experiences including bereavement, lived experience of racial disadvantage and/or current service user representatives).
- Six multidisciplinary staff from wider English maternity system governance organisations including Integrated Care Boards, Local Maternity and Neonatal Systems, the Nursing and Midwifery Council, Maternity and Newborn Safety Investigations, NHS Resolution and a midwifery principal lecturer/lead midwife for education.
- 16 multidisciplinary team staff from across the service including obstetricians, midwives working in a range of roles, for example, community and hospital midwives across the different bands (grades), a midwife sonographer, neonatologists, obstetric and anaesthetists.

All service users were paid agreed NIHR rates for their participation.^49^ The CSG was convened two times in this phase of the project to explore and refine IPTs. Additionally, a multidisciplinary steering committee (MSC) was convened to provide an accountability mechanism while also contributing high-level insights/linkages with policy and horizon scanning. This steering committee comprised two service users (an established service user representative and a founder of a bereavement charity), a methodologist, an obstetrician and a consultant midwife. The MSC met three times over the period that the mechanisms were identified.

## Methods

This paper reports on the development and refinement of IPTs, offering insights into the contexts and mechanisms that may produce positive outcomes across the a priori four domains (previously described) of high-quality maternity care. With embedded mechanisms of effect. The process involved three sub-stages (a) exploring and refining the scope of the review and research question, (b) evidence gathering where data were extracted and synthesised into the IPTs and (c) consultation with the CSG to explore, refine and develop the IPTs.

### Stage 1: refining the scope of the review

**Figure 1:**
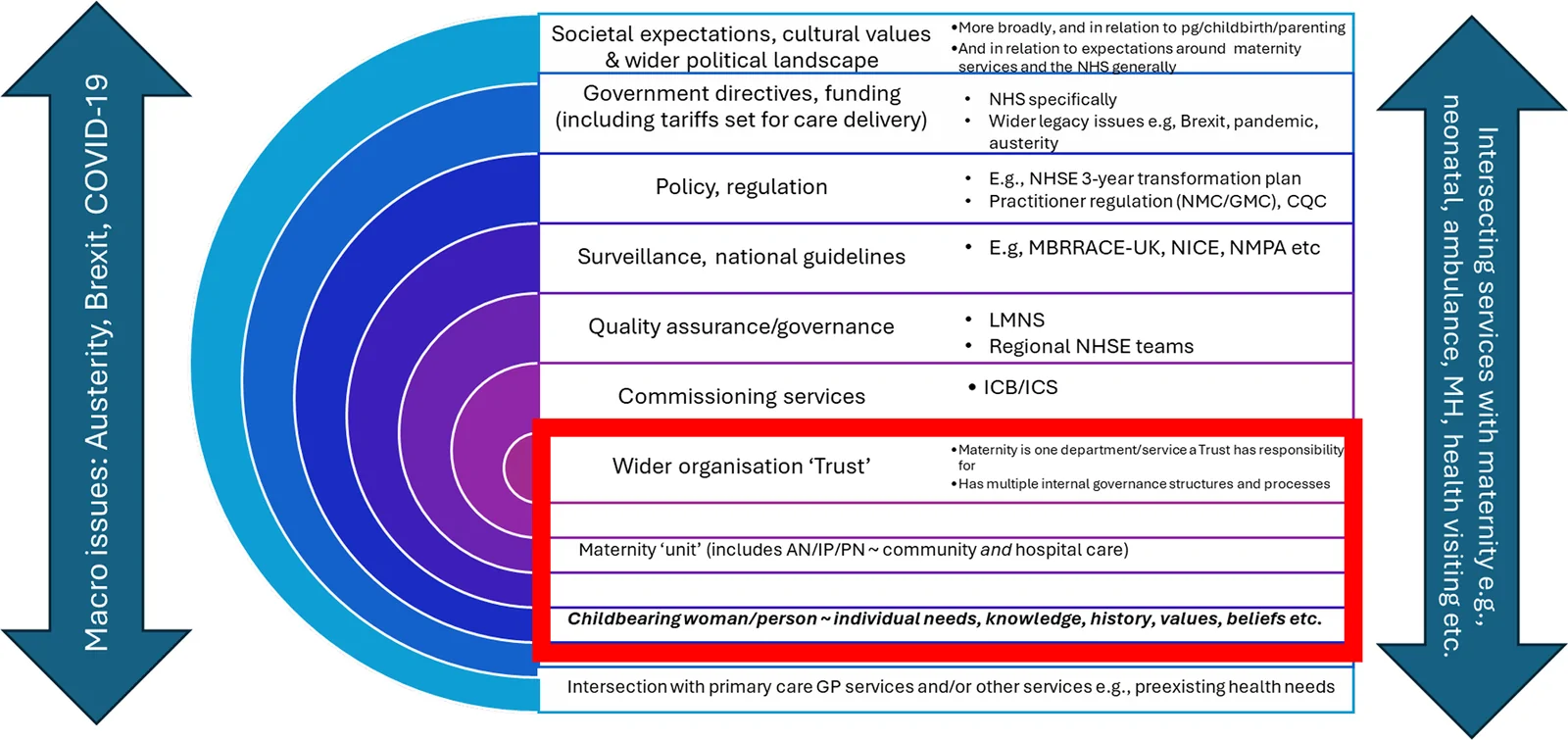
Scope of the review outlines the layers surrounding a maternity organisation in England and the red box indicates the focus for our work. AN, antenatal; CQC, Care Quality Commission; GP, general practitioner; GMC, General Medical Council; ICB, Integrated Care Board; ICS, Integrated Care System; IP, intrapartum; LMNS, Local Maternity and Neonatal System; MH, mental health; MBRRACE-UK, Mothers and Babies: Reducing Risk through Audits and Confidential Enquiries across the UK; NMC, Nursing and Midwifery Council; NICE, National Institute for Health and Care Excellence; NHS, National Health Service; NHSE, National Health Service England; NMPA, National Maternity and Perinatal Audit; PN, postnatal.

The project started with an in-person research team workshop. We divided the day into a series of short presentations followed by discussion and brainstorming activities. The day included a recap on the project aims, objectives and methodology; exploration of our positionality (requiring each of us to report our reflexive position in relation to high-quality maternity care); an overview of the concept of Safety II and the evidence base around ‘quality maternity care’. We created initial sketches of possible programme theories linking our four outcome criteria of interest. These activities informed in-depth discussions to explore and refine the scope/research question of the project. We also mapped out the system levels that might influence individual organisations that deliver maternity services, highlighting from figure 1 the specific focus of our inquiry—the organisational level.

### Stage 2: evidence gathering to develop the IPTs

The next stage involved conducting a focused search of the literature to identify papers and other sources of evidence with sufficient detail and richness to contribute to theory building.^50^ It was designed to identify 10–12 candidate or index papers^48 51^ with which to develop IPTs that explain ‘what works, for whom, in what circumstances’. Additionally, consistent with realist review methodology, searching in this synthesis was purposive and iterative and directed at developing IPT from a sample of literature,^52^ rather than an exhaustive retrieval. Searches were therefore designed to proceed to theoretical sufficiency rather than being updated on a fixed schedule; further searching would be triggered only where residual gaps in the programme theory require additional data.^22 45 50 52^

#### Search strategy

Aligned with a realist approach, our search strategy sought to identify 10–12 candidate papers that were sufficiently rich, diverse and relevant to our focus; to develop tentative explanatory theoretical propositions to answer the research question.^48 50 51^ This approach differs from conventional systematic review-based search strategies which deploy a Population, Intervention, Comparison and Outcome or Population, Exposure and Outcome framework followed by an exhaustive search focusing exclusively on academic papers, requiring regular updated searches. Instead, our approach deployed purposive and iterative searching using a combination of database searches, supplementary searches (including grey literature) and citation chasing to develop theory from a sample of literature.^48 50 51^ Therefore, the search strategy was developed first using an exploratory search via Google Scholar using a conceptual approach to identify papers that focused broadly on ‘high quality maternity care’. We used the following simplified search terms ‘good maternity care’, ‘high quality care pregnancy’, ‘effective AND maternity care’, ‘high-performing AND maternity care’, ‘safe AND maternity care’. As multiple hits were generated, indicating papers could be retrieved with a conceptual approach, we used the same search terms in MEDLINE, where we identified repetitive hits across both databases which were captured and reviewed. Other searches included hand searching, citation chasing and putting out a call to our professional networks. All of this was intentionally carried out iteratively, going back/forth from searches to an initial review of the papers and back again (also see the Screening and selection). See online supplemental file 1.

#### Inclusion/exclusion

Following a realist review methodology, any literature from peer-reviewed journals, grey literature, policy reports, service guidance and public and professional commentary was eligible for inclusion.^50^ Only English literature was included and literature needed to originate from high-income countries with sufficient resources to support best practice. Literature that focused on ‘what works’ at the organisational level and related to all four outcome criteria was prioritised. Research pertaining to specific clinical topics, such as reducing postpartum haemorrhage or perineal damage, etc, was excluded to retain the broader, wider focus of ‘high-quality maternity care’ across the continuum. Mechanisms at a level that is not easily influenced or changed, such as policy, regulation, government directives, funding, societal expectations, cultural values and the wider political landscape, were excluded.

#### Screening and selection

Screening and selection were carried out by ET and CF. The goal in this phase of a realist review is to identify ‘key informant’ or candidate papers.^51^ Rather than undertake a comprehensive search as per conventional systematic reviews, the papers included at this stage were intentionally sought to be ‘highly matched to the review questions’.^51^ Therefore, screening was iterative, going back and forth between the research aims and scope with the papers at hand, and where necessary returning to hand searching. Criteria of *richness, relevance and rigour* (methodological quality of research papers) were applied.^53^ Purposive sampling was used to map potential inclusions against our four criteria.^48 54^ In the first instance, we prioritised literature that appeared to address all four outcome criteria and subsequent documents were included if they addressed at least one. Maximum variation sampling was also employed to include studies and documents across diverse participants, countries, designs and topic foci rather than simply aggregating similar studies.^50 53^ The overall process also involved extensive discussions with the research team and returning to searching as required before finalising inclusions into this stage of the review.

#### Data extraction

ET and CF created, tested and refined a purposely designed data extraction tool using Excel with methodological support from ABo. First, basic study characteristics were extracted, that is, authors, title, type of literature, paper focus, context/setting and the study outcome criteria indicators it related to. Second, the heuristic ‘If, Then, Leading to’ was used to configure data into CMO propositions (see online supplemental file 1).^55^ Here, the ‘if’ relates to context, ‘then’ relates to mechanism and ‘leading to’ relates to the outcomes.^55^ The heuristic was used to label columns in our data extraction tool, and data from each included paper was mapped across these three column headings. Each paper was reviewed in its entirety for data extraction purposes.

#### Data analysis/synthesis

Data analysis and synthesis were carried out in several stages. To manage the volume of data, we excluded the CMOs that did not relate to all four outcome domains simultaneously. Seven CMOs outside of scope (eg, those that related to wider policy or commissioning, etc) were similarly excluded. Second, we used an analogue method where all the included CMOs were combined in a single spreadsheet, printed off and each CMO was cut up individually. Spreading them all out, similar or related CMOs were grouped together by CF and ET. We spent a significant amount of time exploring and discussing these initial groupings, going back and forth cross-checking each group of CMOs had clear alignment. During this phase, we created tentative category labels that illustrated each group of CMOs. Following further immersion and iterative discussions, we came to a consensus of 18 categories with their corresponding CMOs. Third, from these 18 categories (groups of CMOs), we developed IPT statements using the ‘If, Then, Leading to’ heuristic, ensuring we incorporated the nuances of each CMO. The first draft was completed by CF who generated 21 IPT statements. These were refined through discussions with ET, noting some overlap or repetition which allowed some to be collapsed into the other, leaving a final set of 11 IPTs. Following this, feedback from the whole research team was integrated into developing and refining the IPTs further, largely around accessible language and reducing their complexity which was balanced with ensuring we did not lose any important detail from the original CMOs.

### Stage 3: refining the IPTs in consultation with stakeholders

Involving stakeholders in the refinement of the IPTs is a standard component of the realist review methodology.^47 48^ We designed vignettes to make the IPTs relatable for the stakeholders^56^—see online supplemental file 2. Two virtual meetings were convened with the stakeholder group, in which attendees were assigned to a breakout room together with a facilitator (research team member). Within each breakout room, two vignettes were presented alongside key prompt questions to open discussions (eg, ‘How does this resonate (or not) with your own experience? What is needed to make this happen in your setting? Is there anything missing?’). The discussions were recorded (with permission) to ensure we captured everyone’s views adequately. At the follow-up meeting, stakeholders discussed the feedback from the different vignettes. After each meeting, all stakeholders were invited to provide any further feedback on the vignettes and IPTs via a Word document. Eleven stakeholders, including four service users, attended the first meeting and seven stakeholders, including two service users, attended the second one.

Following the stakeholder events, ET and CF collated the discussions and feedback into one document. They then refined the IPTs by working through each point in the collated document (see online supplemental file 3). The updated IPTs were then taken back to the research team for further discussion and review. We also emailed the stakeholder group (online supplemental file 3), so they were aware of refinements made to the IPTs following their input.

## Results

**Figure 2:**
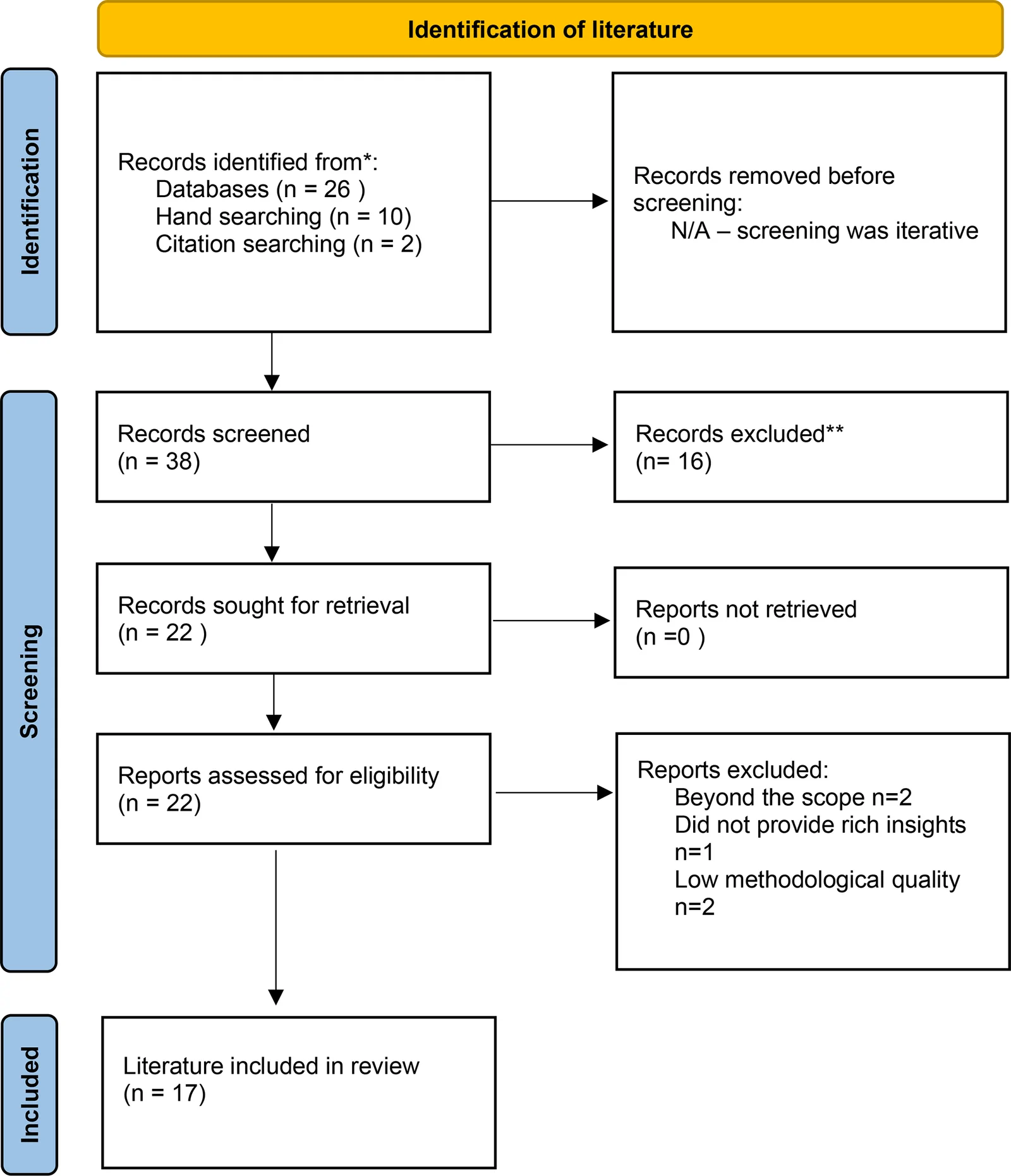
Preferred Reporting Items for Systematic Reviews and Meta-Analyses (PRISMA).

Searches were carried out by ET from October to November 2024, aligned with a realist review approach that sought to generate theoretical propositions via CMO configurations; these were not updated.^22 45 50 52^ The first search yielded 38 candidate papers, of which 22 were selected using the title and abstract. 16 others were removed for reasons including not fitting the scope of the realist review, not providing key information, overlapping authors leading to overlapping views, etc. Next, the 22 papers were obtained and checked at full text. Another five papers were excluded due to not fitting the scope of the research project (eg, beyond the scope of the project, content not specific enough, low methodological quality). Following iterative searching and selection, 17 index papers were included (see figure 2).^57^

### Study characteristics

The included papers comprised systematic reviews n=5, primary research studies n=5, commissioned reports n=2, commissioned national investigation n=1, national evaluation n=1, commentaries n=2 and a local system report n=1. They were from multiple countries and related to current practice as documented between 2016 and 2024. The selected papers were categorised in five different subcategories related to the pre-established outcome criteria for safe, personalised, high quality maternity care; n=3 related to high rates of maternal well-being,^58–60^ n=3 related to high rates of staff well-being,^61 62^ n=2 related to low rates of adverse outcomes,^23 63^ n=2 related to optimal use of resources^1 23 64^ and n=7 were overlapped, with the literature met more than one outcome domain of interest.^65–71^ See online supplemental file 5 for full study characteristics table.

### IPT development

From the included papers, a total of 292 context-mechanism-outcome hypotheses were generated (see online supplemental file 4). Seven were removed as they did not fit our scope and only CMOs related to the organisational level were retained, leaving n=189 CMO configurations. Initially, these configurations were grouped into 18 outcome categories, and 21 tentative IPTs were developed. Following research team feedback, these were synthesised and refined into 11 IPTs and stakeholder feedback facilitated further refinement of these 11 IPTs (see online supplemental file 3).

### Initial programme theories

Box 1 presents the initial IPTs as statements that included the ‘If, Then, Leading to’ heuristic which conveys the context and mechanisms that drive the outcomes of interest (see online supplemental file 6 for the IPTs with citations of the candidate papers they were generated from). Through researcher and stakeholder discussions, it became clear that the IPT related to ‘A culture of equitable care’ was viewed as crucial and should underpin any and all services that provide maternity care.

Box 1 **Initial programme theories (IPTs)**

**IPT conceptual label and content**

**A listening, hearing, learning and acting culture: responding to staff and service user insights**

IF organisations embed and endorse a central and systematic approach (eg, investment in the required time and resources, central data intelligence gathering and sharing) to a listening, hearing, learning and acting culture* inclusive of service users and staff alike THEN staff are more likely to implement a shared sense of ownership and responsibility for continual service improvements THEN this creates a responsive, transparent, constructive, supportive mechanism for quality improvement LEADING TO sustained high-quality care that is adapted to the population and individual needs in which staff and women feel respected and heard, particularly in case of adverse events.

*Meaning: to listen and act on what is heard, troubleshooting, pre-empting adverse events and disseminating lessons learnt. Fostering robust accountability to the local community.

**The organisation of maternity care is centred on women’s values and individualised/personalised needs by resourcing staff effectively**

IF maternity services organise and invest in high-value care from the perspectives of those using the services*, where care is coordinated by a named professional who works within an aligned multi-disciplinary team and all staff have the appropriate support and resources** to provide relational care and maternal choice*** across the spectrum THEN service users are able to participate in informed decision-making in a meaningful way while staff feel safe and able to discuss and justify personalised care plans (eg, care that is ‘out of guidance’) LEADING TO higher quality care for all women, seamless pathways, better outcomes and experiences for women and staff and a better use of resources.

*Evidence-based and personalised, high-value maternity care across the continuum and across all settings (which are appropriately and equitably resourced, ringfenced and accessible* to all women). **All maternity staff are enabled to develop meaningful and trusting relationships with women, provide accessible evidence-based information clearly and impartially, listen closely to questions, preferences and decisions communicated by maternity service users, and respond to them in a clear and impartial way while mediating full scope safety and this care is led and coordinated by a lead care provider. ***Maternal choice is in place, both for the support of physiological processes, and for interventions where they are needed or wanted.

**High-quality maternity adopts a broader definition of safety (beyond just physical safety)**

IF maternity organisations, leadership and staff adopt a perspective of safety that includes both technical/clinical safety and psychological, emotional and cultural safety as defined by all stakeholders involved in maternity care which is embedded throughout all governance structures intelligent data gathering*, and resourcing, THEN all stakeholders adopt and champion a broad perspective on safety, creating a clinically, emotionally, culturally and psychologically safe environment for all LEADING TO **optimal care delivery across all four domains**.

*Triangulation of data gathering (outcomes and service user feedback (in the short and long-term), staffing levels, resources, time, flexibility, etc).

**Visible and supportive leadership enhancing the workplace and safety culture**

IF knowledgeable and skilled maternity leaders manage hierarchies flexibly and strategically*, role model, while being highly visible on the shop floor, supporting and setting clear standards for what is considered appropriate actions and decisions (including safe escalation routes), explaining how and why, allowing colleagues to develop their skills through reflection-in-action*** THEN staff feel supported and enabled to work autonomously, to develop their skills within the full scope of their practice in united, passionate, coordinated and stable teams THEN actions are taken that are based on the requirements of the situation rather than on hierarchies protecting organisational interests, LEADING TO better outcomes and experiences for women and staff, and a better use of resources.

*Achieving a balance between supporting the learning and allowing staff to make autonomous decisions. **Hierarchies can be mobilised when needed to clarify social norms and behavioural standards but can be muted where not required. ***Aligned with psychological safety and attendance to the three core needs of autonomy, belonging and contribution.

**All maternity care systems, processes, procedures and care are grounded in evidence-based frameworks**

IF the organisation employs ongoing ‘best practice’* evidence-based frameworks to support all systems and processes, including governance** led by appropriately skilled professionals, THEN an evidence-informed, inclusive, collaborative and participative approach can flourish encompassing full-scope safety, effectiveness and person-centredness LEADING TO the adoption, delivery and sustainability of evidence-based person-centred care in which providers can make the right decisions at the right time based on women’s choices and needs.

*‘Best Practice’: Evidence-based practice which consists of the triad of women’s needs, best available clinical evidence and clinical expertise. **For example, for guideline development, the team should have appropriate research (systematic review) skills (eg, a fundamental valuing and reinforcement that all these things should be done by people with the appropriate knowledge and skills).

**The system and workplace environment supports optimal clinical performance (operational fitness)**

IF organisations design, implement and continually assess/review their working environments including systems, processes, structures, equipment, IT and facilities are optimised and aligned with human factors/ergonomics* and Safety II principles and deploy intelligent data gathering (soft and hard) to manage system risk THEN the safety of the ‘system’ is enhanced/optimised with features of increased flexibility, preparedness and responsiveness to actual events/needs of the service (rather than reactive) THEN with increased staff well-being, decreased cognitive burdens enabling staff to work to their full scope of practice (eg, using clinical skills not administrator skills) LEADING TO optimal care delivery **across all four domains**.

*Psychological safety and attendance to the three core needs of autonomy, belonging and contribution.

**The organisation and its systems are adapted to staff needs**

IF maternity care organisations ensure minimum standards for basic facilities and staff support* for all aligned with psychological safety and attendance to the three core needs of autonomy, belonging and contribution THEN this improves working conditions, optimises health and well-being for staff (as they feel valued, supported and empowered), LEADING TO optimal care delivery **across all four domains**.

*Examples of basic facilities and staff support: places and time to rest and sleep, access to nutritious food and drinks, the tools needed for staff to do their job (eg, parking, MDT training, (clinical) support, implement educational packages, respect, autonomy, cultural safety, consideration of work schedules based on realistic forecasting and demands that account for fatigue, supports safe shift swapping and enables breaks consistently and also providing easily accessible workplace tools to support clinical activity, for example, Wi-Fi, mobile desktops and evidence-based clinical resources, pathways and guidelines that help staff to do the right thing, a practical layout of the ward, etc).

**Leadership drives a high-quality service by attending to the triad of safety, service user experience and clinical effectiveness**

IF maternity leaders ensure that all aspects of the quality triad—safety, patient experience/person-centredness and clinical effectiveness are in place by authentically and proactively involving all staff and a wide range of service-users and acting on their views*; working with the local community and voluntary sector organisations THEN this facilitates strong, compassionate and courageous leadership and management enabling the MDT to work well across the system LEADING TO a culture of caring, choice and a strong service-user voice.

*For example, intelligent data gathering and sharing is triangulated with all stakeholders to ensure accountability of senior teams

**Responsiveness to ongoing workload and acuity assessments accounting for high-value care and the different workplace contexts**

IF organisations implement and sustain a programme to review workload in their organisations that takes account of the specific context and work that is done in different areas of the service (with appropriate skill mix) and the nature of high-value care from the perspectives of those using the service, THEN the service can be adequately and appropriately resourced across the board, teams are protected to provide their services while also tackling excessive work demands on staff LEADING TO sustainability in delivering safe, high-value, high-quality care and meeting core staff needs so service users receive high-quality, safe and compassionate care.

**Resource management for meaningful choice provision and optimal care with seamless care pathways for all service user needs**

IF organisations implement and sustain evidence-based, responsive and personalised, high-value maternity care across the continuum and across all settings (which are appropriately and equitably resourced, ringfenced and accessible* to all women) THEN seamless pathways exist for all women (eg, both a ELCS AND homebirth pathway are available), meeting individual needs while addressing inequalities THEN women and babies are offered the right care at the right time LEADING TO improved outcomes, improved well-being, optimal use of resources.

*Meaning: assurance of geographical, social and economic access for all women.

**A culture of equitable care underpins all other IPTs**

IF the maternity service engages in meaningful ways with marginalised and minoritised groups, including socially disadvantaged and the global majority and commits to co-producing anti-racist policies and practices* while engaging local voluntary groups and the local community sector and providing cultural safety training for staff THEN the culture of the organisation and frontline staff perpetuate inclusive, respectful and ethical behaviours LEADING TO staff and service users from the Global Majority communities feeling and being safer and having better experiences, improved staff work experience.

*For example, culturally appropriate interpretation and/or doula services, ensuring continuity.

## Discussion

### Summary of findings

This first phase of our realist review that explored the contexts and mechanisms that drive high-quality maternity care outcomes specifically at the organisational level of maternity service delivery drew on 17 sources of international literature, generating 11 IPTs. These IPTs covered the organisation of maternity services based on high-value care from the perspectives of service users, including broadening notions of ‘safety’ and resource management for seamless care pathways attending to individualised needs; embedded systems to foster authentic listening, learning and acting cultures; systems that are grounded within evidence-based frameworks and which have ‘operational fitness’—adaptable and responsive to workloads and the needs of staff; and the necessity of visible and proactive leadership. Collectively, these IPTs convey the contexts or circumstances required to stimulate key mechanisms that facilitate the delivery of safe high-quality maternity care from service user, staff and a systems perspective. For example, reducing staff cognitive burden and supporting staff well-being measures provides an enabling environment to provide high-quality care to meet the needs of individual service users. Overall, the four outcome criteria that underpinned our definition of high-quality maternity care are strongly supported by the current global evidence base. The realist approach was challenging, particularly when deciding which papers to include, and how to limit the potentially very large number of CMOs and therefore IPTs that could be generated. Despite this, the process generated a set of IPTs with high resonance across our broadly constituted stakeholder group.

Broadly, the IPTs encompassed a ‘both-and’ philosophy, where both clinical excellence and values-based relational care were equally expressed. This dual perspective was true for both service users and for the staff working environment. This was important to capture as a recent study found notions of ‘quality maternity care’ differed among various stakeholders, with some perspectives centring organisational safety features (eg, adverse outcomes) and others centring values-based features such as relational continuity of carer.^72^ It is thus important that IPTs bridge both perspectives, as without a values-based approach, women and birthing people may opt away from the maternity service offered.^73 74^ Furthermore, we found crucial data to advance the everyday understandings of ‘organisational safety’ through the development of an IPT that indicates ‘high-quality maternity care adopts a broader definition of safety’. This IPT addresses limitations to the safety agendas within many maternity services that tend to hyperfocus on narrowed clinical outcomes, often at the expense of important issues such as cultural safety and emotional/psychological safety.^66 75^ Emotional safety is being viewed as a core driver towards service user engagement with maternity care and their outcomes and overall experiences of maternity care—reflecting the wider literature.^76–79^ For example, each English public inquiry to date^4–8^ and the most recent Amos interim report^10^ cite key issues of women and birthing people not being listened to, their concerns not taken seriously and their needs not met. These issues were reported as instances of poor-quality maternity care and in some situations were deemed as the cause of adverse outcomes.^10^ These demonstrate the importance of the so-called ‘soft’ skills and emotional safety being central to the delivery of high-quality maternity care to produce positive biopsychosocial outcomes.

Additionally, global evidence suggests there is a growing concern about poor cultural safety; for example, in countries such as England where the disparity between Black and white women’s outcomes remains persistently poor highlights the importance of resolving cultural safety issues.^37 80^ Therefore, our work strongly asserts that ‘a culture of equitable care’ must be embedded throughout maternity systems to address the multiple disadvantages that many women and birthing people face. High-quality maternity care, particularly models such as Midwifery Continuity of Carer have been shown to mitigate against structural disadvantages that tend to increase poorer outcomes.^81–83^ Advancing this evidence, our IPT situates that equitable care must be embedded and permeated throughout the maternity system via proactive, meaningful engagement with the local communities that are most disadvantaged.^84 85^ Such committed practice is likely a core mechanism to generate an equitable culture within an organisation. In this regard, the outcomes serve both service users and staff, where those from the Global Majority also face racial discrimination within the workplace.^86^

### Implications

While other realist reviews have explored key components of maternity care delivery, for example, telehealth provision,^87^ midwifery continuity of carer^81 88^ and interventions in LMICs,^89^ this is the first to explore the broad concept of ‘high-quality maternity care’ delivery specifically at the organisational level. Through this work, we hypothesise that sites that report higher than average staff and service user well-being, better than average clinical and psychological outcomes for service users, and judicious use of resources will be activating or ‘firing’ some or all of these mechanisms. In regard to the UK context, given numerous inquiries and reviews have been conducted into ‘failing’ services with a national investigation in progress and given the lack of progress in improving maternity care in England (and even the reversal of progress in some aspects), we would argue that it is time to take a different approach to improving maternity care. Rather than focusing only on the Safety I approach of what goes wrong, it is critical to also learn from the Safety II analysis of what goes right. The IPTs developed in this project could form a basis for initiating such an approach.

### Strengths and weaknesses

Taking a realist approach, while methodologically complex, does offer meaningful insights that account for complex adaptive systems such as maternity services. Realist thinking seeks to understand the context(s) that ‘fire’ the mechanisms to produce the outcomes of interest.^45^ Therefore, realist syntheses are likely to generate more ‘real world’ applications than other types of reviews. However, as with any qualitative reviews, there is a risk of under- or over-interpreting the data—we mitigated this concern through our stakeholder engagement activities and will seek to ‘test’ the IPTs with the wider literature in the next phase of the review. These could also be ‘tested’ via empirical research using realist evaluation methodology to capture contemporary maternity care delivery within its current context. There was also a risk of neglecting contextual insights that would influence these IPTs as we excluded literature exploring mechanisms at the system or policy level. This was an intentional decision to ensure this review retained international relevance (system/policy levels will differ) and was achievable within the funded timeframe. This should be addressed during a realist evaluation where empirical evidence is captured in specific country contexts, and these components can be explored and integrated to generate causal insights and programme theories. Furthermore, the decision to exclude CMOs that did not relate to all four outcome domains during this phase may have constrained theory development; however, as our research question was based on the coalescence of these four domains, we felt this was an appropriate decision. Additionally, the studies used to develop these IPTs are globally relevant (to high-income contexts); future work should test against literature from languages other than English and from other cultural contexts. However, based on the current literature on respectful maternity care we would expect that many of the IPTs would resonate with this broader literature.

## Conclusion

This paper reported on the first phase of a realist review exploring the mechanisms of high-quality maternity care at the organisational level of service delivery. The 11 IPTs that were developed with stakeholder engagement activities offer crucial insights into the context and mechanisms which may produce outcomes related to staff and service user well-being, clinical and psychological outcomes for service users and judicious use of resources. This phase provides the foundation for future realist research, either as the second phase of a realist review or an empirical evaluation. Given we used global literature within high-income contexts, the IPTs reported here could extend to other country contexts. Moreover, the premise of learning from ‘high-quality’ maternity services, as opposed to failing services, offers a novel way to reconsider the challenges currently faced by global maternity care.

## Supplementary material

10.1136/bmjopen-2026-119351 online supplemental file 1

10.1136/bmjopen-2026-119351 online supplemental file 2

10.1136/bmjopen-2026-119351 online supplemental file 3

10.1136/bmjopen-2026-119351 online supplemental file 4

10.1136/bmjopen-2026-119351 online supplemental file 5

10.1136/bmjopen-2026-119351 online supplemental file 6

## Data Availability

Data are available on reasonable request.
