# Supplementary material for: Understanding the contexts and mechanisms that drive high-quality maternity care outcomes within organisations: generating 11 initial programme theories as phase 1 of a realist review
Source: BMJ Open. 2026 Aug 31;16(8):e119351. doi: 10.1136/bmjopen-2026-119351 (PMC13536141; doi:10.1136/bmjopen-2026-119351)
Supplement: Supplementary file 1 [file bmjopen-16-8-s002.pdf]

IPT Vignettes

Theme: A listening, hearing, learning and acting culture: responding to staff and service user insights

IF organisations embed and endorse a systematic approach (e.g. investment in the required time and resources) to a listening, hearing, learning and acting culture* including data intelligence gathering inclusive of service users and staff alike THEN staff are more likely to implement a shared sense of ownership and responsibility for continual service improvements LEADING TO a responsive, transparent, constructive, supportive mechanism for quality improvement LEADING TO sustained high-quality care that is adapted to the population and individual needs in which staff and women feel respected and heard, particularly in case of adverse events

* Meaning: to listen and act on what is heard, troubleshooting, pre-empting adverse events.

Vignette

I am a healthcare professional in a maternity service. My team and I are actively encouraged to speak up about what goes well and what does not go well within the unit. Our leadership team genuinely listens to us and provide various ways for us staff and service users to provide feedback throughout the pregnancy, birth and postnatal journey. All actions as a result of this feedback are reported back to us and the families in different ways making us feel listened to and heard. We as staff participate and contribute to improvement projects which has created a culture of support and respect, also helping us to anticipate and act on potential issues before they become bigger problems. We pride ourselves in being flexible and able to respond to surfacing problems promptly, enabling us to continually provide great care.

Theme: The organisation of maternity care is centred on women's values by resourcing staff effectively

IF maternity organisations direct their resources toward in high-value care centred on women's values from the perspectives of those using the services*, where care is coordinated by a named professional who works within an aligned multi -disciplinary team and all staff have the appropriate support and resources** to provide relational care and maternal choice*** across the spectrum THEN service users are able to participate in informed-decision making in a meaningful way while staff feel safe and able to discuss and justify personalised care plans (e.g. care that is 'out of guidance') LEADING TO higher quality care for all women, seamless pathways, better outcomes and experiences for women and staff and a better use of resources.

* Evidence-based and personalised, high value maternity care across the continuum and across all settings (which are appropriately and equitably resourced, ringfenced, and accessible* to all women)

** All maternity staff are enabled to develop meaningful and trusting relationships with women, provide accessible evidence-based information clearly and impartially, listen closely to questions, preferences and decisions communicated by maternity service users, and respond to them in a clear and impartial way while mediating full scope safety & this care is led and coordinated by a lead care provider.

*** maternal choice is in place, both for the support of physiological processes, and for interventions where they are needed or wanted.

** maternal choice is in place, both for the support of physiological processes, and for interventions where they are needed or wanted.

Vignette

I am a pregnant woman receiving maternity care. My care is coordinated by one clinician with who I have been able to build a relationship with and who I can contact in case I have questions or concerns. I am well informed by all healthcare providers of the multidisciplinary team that I meet along my pregnancy journey and I know who to contact if I have a problem. I feel they are all taking the time to listen to me. My providers always give me a range of options, in an impartial or non-judgmental way, while considering what makes me feel safe. This makes me feel I am in good hands as I know someone is looking out for me and care is adapted to me as an individual.

Theme: High-quality maternity adopts a broader definition of safety (beyond just physical safety)

IF maternity organisations, leadership and staff adopt a perspective of safety that includes both technical/clinical safety and psychological, emotional and cultural safety as defined by all stakeholders involved in maternity care which is embedded throughout all governance structures intelligent data gathering*, and resourcing, THEN all stakeholders adopt and champion a broad perspective on safety, creating a clinically, emotionally, culturally and psychologically safe environment for all LEADING TO all four outcome indicators.

Vignette

I am a healthcare professional and work in an organisation that visibly recognises that safety is a bigger concept than physical safety for both service users and staff. Our leadership teams have emphasised and reinforce the importance of ensuring that women and birthing people feel safe (emotionally, psychologically, culturally) and have their individual needs met throughout their maternity journey. I feel equipped with the right skills and support to provide safe clinical care that centres someone's individual needs as the systems and resources are in place that support this. We pride ourselves in the great physical and psychological outcomes we achieve and the feedback we get from women and their families.

Theme: Visible and supportive leadership enhancing the workplace and safety culture

IF knowledgeable and skilled maternity leaders manage hierarchies flexibly and strategically*, role model, while being highly visible on the shop floor, supporting and setting clear standards for what is considered appropriate actions and decisions (including safe escalation routes), explaining how and why, allowing colleagues to develop their skills through reflection-in-action THEN staff feel supported and enabled to work autonomously, to develop their skills within the full scope of their practice in united, passionate, coordinated and stable teams LEADING TO actions taken that are based on the requirements of the situation rather than on hierarchies protecting organisational interests, LEADING TO better outcomes and experiences for women and staff, and a better use of resources.

* Achieving a balance between supporting the learning and allowing staff to make autonomous decisions.

** hierarchies can be mobilised when needed to clarify social norms and behavioural standards but can be muted where not required

Vignette

I am a clinician working at a maternity service. Our team is supported to make the best use of the appropriate expertise depending on what the situation calls for meaning we have a flexible approach to leadership. This is supported by the designated leadership team who are visible and approachable on the ward. They are great role models for the team, helping us out where needed and demonstrating good practice but also trusting us in practicing the full scope of our profession. We feel safe to reflect on care given (what went well and what could be improved upon). This has led to a cohesive, responsible and passionate team in which individuals can safely develop their skills while also providing great maternity care.

Theme: All maternity care systems, processes, procedures and care are grounded in evidence-based frameworks

IF the organisation employs ‘best practice’ evidence-based frameworks to support all systems and processes including governance (e.g., guideline development, guideline developers being at the forefront of guideline methodology, quality improvement), THEN an evidence-informed inclusive, collaborative, and participative approach can flourish encompassing full-scope safety, effectiveness, and person-centredness LEADING TO the adoption, delivery and sustainability of evidence-based person-centred care in which providers can make the right decisions at the right time based on women’s choices and needs.

Vignette

I am a healthcare professional at a maternity service. In my organisation, all clinical staff and individuals in leadership roles understand the importance of evidence-based practice. This includes evidence-based clinical practice, governance procedures, quality improvement approaches, change implementation and participatory approaches with service users. Guidelines/systems are created and agreed upon within the multidisciplinary team alongside

service users and using the latest high-quality evidence-based knowledge by clinicians who have solid methodological knowledge and skills. I feel the system in place provides me with everything that I need to make the right decisions at the right time, based on women's choices and we are able to provide excellent, safe, effective, person-centred care.

Theme: The system & workplace environment (operational fitness) supports optimal clinical performance

IF organisations design, implement and continually assess / review their working environments including systems, processes, structures, equipment, IT and facilities are optimised aligned with human factors/ergonomics* and Safety II principles and deploy intelligent data gathering (soft and hard) to manage system risk THEN the safety of the 'system' is enhanced/optimised with features of increased flexibility, preparedness and responsiveness to actual events/needs of the service (rather than reactive) LEADING TO increased staff wellbeing, decreased cognitive burdens for staff and enabling staff to work to their full scope of practice (e.g., using clinical skills not administrator skills) LEADING TO operational 'fitness' within an organisation and optimal care delivery where staff are enabled to do the right thing consistently, better teamwork and job satisfaction, retention of the workforce and high-quality care provision improving outcomes and experiences of care/workplace.

* Psychological safety and attendance to the three core needs of autonomy, belonging and contribution

(e.g. Examples of basic facilities and staff support: places and time to rest and sleep, access to nutritious food and drinks, the tools needed for staff to do their job (e.g. MDT training, (clinical) support, implement educational packages, respect, autonomy, consideration of work schedules based on realistic forecasting and demands that accounts for fatigue, supports safe shift swapping and enables breaks consistently and also providing easily accessible workplace tools to support clinical activity e.g., Wi-Fi, mobile desktops and evidence-based clinical resources, pathways and guidelines that help staff to do the right thing, a practical lay-out of the ward, etc.)

Vignette

I am a healthcare professional at a maternity service and when I go to work, mostly things work smoothly and practically. E.g. when I login to the computer at work, it opens promptly, and software used is instinctive. I easily can access and get an overview of the most important medical, obstetric, and social information of the women I look after. All medicines and equipment are stocked and stored in a logical, economical and practical manner, so I don't lose time. I feel I can easily do my job, especially in emergency situations in which I quickly need to get information and the necessary material to act. I can focus on the important clinical tasks at hand to improve outcomes while developing and maintaining positive relationships with women and birthing people enhancing their experiences while also looking after myself.

Theme: A culture of equitable care

IF the maternity service engages in meaningful ways with marginalised and minoritised groups, including socially disadvantaged and ethnic minoritised communities and commits to co-producing anti-racist policies and practices while providing cultural safety training for staff THEN the culture of the organisation and frontline staff perpetuate inclusive, respectful and ethical behaviours LEADING TO improving the quality, safety and inclusiveness of services for these groups LEADING TO staff and service users from the Global Majority communities feeling and being safer and having better experiences, improved staff work experience.

Vignette:

I am a woman of an ethnic minority background. One of the leaders in my community has helped writing and implementing anti-racist policies and practices and advised me to book my pregnancy at this service. When receiving maternity care in this unit, I felt that the services were adapted to my individual needs. For instance, when I went for the female genital mutilation appointment, there was someone from my cultural and ethnic background present, who also spoke my native language. Furthermore, I was given leaflets in my native language so I could understand the risks and benefits of the screening and treatments offered to me. Staff were very respectful and always maintained my dignity. This made me feel respected, understood, and overall, very safe.

Theme: The organisation and its systems are adapted to staff needs

IF maternity care organisations ensure minimum standards for basic facilities and staff support* aligned with psychological safety and attendance to the three core needs of autonomy, belonging and contribution THEN this improves working conditions, optimises health and wellbeing for staff (as they feel valued, supported and empowered), LEADING TO optimal care delivery where staff are enabled to do the right thing consistently, better teamwork and job satisfaction, retention of the workforce and high-quality care provision.

* Examples of basic facilities and staff support: places and time to rest and sleep, access to nutritious food and drinks, the tools needed for staff to do their job (e.g. MDT training, (clinical) support, implement educational packages, respect, autonomy, consideration of work schedules based on realistic forecasting and demands that accounts for fatigue, supports safe shift swapping and enables breaks consistently and also providing easily accessible workplace tools to support clinical activity e.g., Wi-Fi, mobile desktops and evidence-based clinical resources, pathways and guidelines that help staff to do the right thing, a practical lay-out of the ward, etc.)

Vignette

I am a healthcare professional working at a maternity service. Within my organisation I feel enabled to practice the full scope of my profession while I also feel very supported and respected by the multidisciplinary team. The workload is manageable, and my breaks are guaranteed while having access to a comfortable break room, nutritious food and drinks. I

feel supported and empowered by my team and manager to provide good care and furthermore have a great work-life balance.

Theme: Leadership drives a high-quality service by attending to the triad of safety, service user experience and clinical effectiveness

IF maternity leaders ensure that all aspects of the quality triad – safety, patient experience / person-centredness and clinical effectiveness are in place by authentically involving service-users and acting on their views THEN this facilitates strong, compassionate, and courageous leadership and management enabling the MDT to work well across the system LEADING TO a culture of caring, choice, and a strong service-user voice.

Vignette

I am a maternity leader, and my main goal is to ensure that all women and babies receiving care at this unit receive care that is safe, effective and adapted to their individual needs. This I do by authentically listening to-, involving, and acting on the feedback given during the stakeholder meetings that I regularly organise. I am aware of what goes on at the frontline of care and ensure that all staff knows the importance of the voices of women and families. This means that together, we continually can improve our services.

Theme: Responsiveness to ongoing workload and acuity assessments accounting for high-value care and the different workplace contexts

IF organisations implement and sustain a programme to review workload in their organisations that takes account of the specific context and work that is done in different areas of the service and the nature of high-value care from the perspectives of those using the service, THEN the service can be adequately and appropriately resourced across the board, teams are protected to provide their services while also tackling excessive work demands on staff LEADING TO sustainability in delivering safe, high-value, high-quality care and meeting core staff needs so service users receive high-quality, safe, and compassionate care.

Vignette

I am the director of a maternity service. I have implemented an evidence-based tool that measures all the activities that are happening in the different services, which considers the different contexts. This has enabled me to adequately and appropriately resource the maternity services, so women receive the right care at the right time across the board and no services are deprived of staff or resources. Staff feel happier as their workloads are manageable and they feel their work is appreciated. Women receive the care that is high value to them. Services are sustainable and meet everyone's needs.

Theme: Resource management for meaningful choice provision and optimal care with seamless care pathways for all service user needs

IF organisations implement and sustain evidence-based and personalised, high value maternity care across the continuum and across all settings (which are appropriately and equitably resourced, ringfenced, and accessible* to all women)

THEN seamless pathways exist for all women (e.g., both a ELCS AND homebirth pathway are available), meeting individual needs whilst addressing inequalities LEADING TO women and babies being offered the right care at the right time LEADING TO improved outcomes, improved wellbeing, optimal use of resources.

* Meaning: assurance of geographical, social, and economic access for all women.

Vignette

I received care at a maternity service that provided/presented (me with) a lot of scientific knowledge and choices adapted to my needs throughout my care journey. All four places of birth (home, alongside midwifery unit, freestanding midwifery unit, and labour ward) were presented as an option and I also was told that I could request a caesarean section if I wanted to. I was looked after well on the birth centre but due to complications I was advised to be transferred to labour ward, which I agreed to. Transfer was seamless due to great communication and teamwork of all staff involved. Care kept on evolving around my needs and wishes, no matter which ward I was on, or which staff member was looking after me and my baby. I had a great experience as I received the right care at the right time.
