# Supplementary material for: Understanding the contexts and mechanisms that drive high-quality maternity care outcomes within organisations: generating 11 initial programme theories as phase 1 of a realist review
Source: BMJ Open. 2026 Aug 31;16(8):e119351. doi: 10.1136/bmjopen-2026-119351 (PMC13536141; doi:10.1136/bmjopen-2026-119351)
Supplement: Supplementary file 2 [file bmjopen-2026-119351.reviewer_comments.pdf]

PEER REVIEW HISTORY

BMJ Open publishes all reviews undertaken for accepted manuscripts. Reviewers are asked to complete a checklist review form and are provided with free text boxes to elaborate on their assessment. These free text comments are reproduced below.

ARTICLE DETAILS

Title (Provisional)

Understanding the context and mechanisms that drive high-quality maternity care outcomes within organisations: Generating 11 Initial Programme Theories through realist review.

Authors

Feeley, Claire; Thaels, Ellen; Booth, Andrew; Quashie, Michelle; Pradhan, Farrah; Greenstock, Kate; Burnett, Alicia; Walker, Kate; Chang, Yan-Shing; Prescott, Gordon; Rigby, Katrina; Downe, Soo

---

VERSION 1 - REVIEW

**Reviewer**	**1**
**Name**	**Eaton, Georgette**
**Affiliation**	**University of Oxford, Nuffield Department of Primary Care Health Sciences**
**Date**	**15-Apr-2026**
**COI**	**None**

This manuscript demonstrates a strong conceptual understanding of realist methodology and incorporates several important elements of realist review. However, while the study aligns broadly with the intent of RAMESES guidance, there are several areas where the application and reporting of realist methodology could be strengthened to improve transparency, explanatory depth, and reproducibility.

- The manuscript clearly states a realist aim, namely to understand “what works, for whom, in what circumstances.”

- The use of “If–Then–Leading to” structures shows an appropriate attempt to operationalise CMO logic.

-The search strategy is purposive and iterative, which is appropriate for a realist review.

-The inclusion of grey literature is consistent with realist methodology and supports the use of rich, relevant evidence.

-Stakeholder involvement is well described and appears to have been meaningfully incorporated into the review process.

- The IPTs are clearly presented and easy to follow.

- The synthesis makes a credible attempt to connect contexts, mechanisms, and outcomes.

- The manuscript would benefit from greater clarity around mechanisms. In several places, mechanisms appear descriptive rather than generative, and some terms read more like organisational features (for example, leadership or teamwork) than underlying causal mechanisms such as reasoning, responses, or resources.

- The distinction between context and mechanism is sometimes blurred, particularly where organisational features appear to be used in both domains.

- The search process needs to be reported more transparently. Greater detail is needed on how searches evolved and how inclusion and exclusion decisions were made.

-The exclusion of mechanisms at policy or system level is potentially problematic for realist work, as such mechanisms often shape organisational outcomes and may be highly relevant to the review question.

- The decision to exclude CMOs that did not relate to all four outcome domains may have constrained theory development and removed potentially important explanatory material.

- The analogue cut-and-group process is acceptable, but it needs to be described in more detail to allow readers to follow the analytic logic.

- The role of stakeholders in the refinement process would benefit from greater clarification. It is not always clear whether they were validating, refining, or generating theory, or how differing views were handled.

- The manuscript would be stronger if it moved further beyond description and offered more explanatory synthesis, including patterns across studies, tensions or contradictions, and comparison of contexts where mechanisms appeared to work differently.

**Reviewer**	**2**
**Name**	**Gadsby, Erica**
**Affiliation**	**University of Stirling**
**Date**	**05-Jun-2026**
**COI**	**None**

In my opinion, this is a valuable contribution to the literature, clearly explained and robustly executed. Below are a few points for clarification/amendment:

Abstract says this paper reports the first phase of a review. But it doesn't explain the link to the next phase. P26 line 48-49 says that next phases is to 'test' the IPTs with the wider literature – interesting to consider how that will be done, given the complexity of the IPTs developed. Is testing with more literature the most logical next step? What might that look like?

Strengths and limitations: 'the approach is methodologically complex making them challenging to conduct' – wording needs amending.

Some of the references seem wrongly attributed. For example, refs 22 and 44 are both used to support the explanation of what a realist review is (for). But neither of these refs relate to realist methodology – one is a qualitative systematic review, one is 'England N Maternity Transformation Programme'.

Some typos that need ironing out at proofing stage.

P10 line 8 – mentions papers were excluded for low methodological quality, but this isn't normally a criteria in a realist review. And it doesn't sit with the main criteria of richness and relevance. How was methodological quality assessed, and was a consistently reviewed across all documents?

P10 lines 18-33 – summary of included papers appears twice, at beginning and end of paragraph, but with small differences.

The lengthy supplementary docs were very difficult to engage with/interpret in the format presented.

Reviewer 3

Name Abrams, Ruth

Affiliation University of Surrey, School of Health Sciences, Faculty of Health and Medical Sciences

Date 03-Jul-2026

COI None

Thank you or the opportunity to review the manuscript titled: Understanding the mechanisms that drive high-quality maternity care within organisations: Generating 11 Initial Programme Theories through realist review. It is pleasing to see an alternative narrative around maternity care, and I welcome this review. Overall it is very well written, though at times it does read like a funding proposal. That aside, the paper is rigorous and clear, and I hope the authors find this feedback helpful.

Abstract: The abstract is very clear and presents the rationale, methods and findings very clearly.

Intro: This is an excellent introduction, one which clearly sets out the landscape, problem and rationale. A minor point is that you mention drawing on international literature for this study. However I did wonder at this point whether this meant all countries including low to middle income, who have very different circumstances surrounding maternity care. Could the authors clarify in the introduction, which international literature they mean/ any boundaries around this up front?

Methods: Again this is all very clearly written, accurate and appropriate. However, can the rationale for only describing mechanisms be more clearly articulated in the paper. It is not typical to see mechanisms discussed in isolation, as part of the realist approach, largely because they do not operate in isolation, being instead triggered by their relational context. Furthermore, when it comes to your findings- you articulate the statements in full so it feels like you have done yourselves a slight disservice by saying you've only got mechanisms, when actually you've done a lot more work- you have an entire IPT comprising 11 if-then-leading to statements. I'd strongly suggest framing your work in this way, to reflect it's comprehensiveness.

Stakeholder involvement is very clearly described and this is welcome as it is so often a part of realist work that remains under-described. Page 7, line 36 there is a typo here. Page 9, line 13, also a typo.

My only concern with this section, which is repeated throughout as well is that you state you are using the heuristic, if-then-leading to. Typically in realist approaches, it is if-then-because, with the 'if' corresponding to context, then 'then' corresponding to the outcome, and the 'because' corresponding to the mechanism. Here you are using your 'then' to describe the mechanism, it would appear. I think this needs to be made explicit.

Findings

There are sentences in the study characteristics paragraph that are repeated.

Suggest the table of document characteristics is moved to your supplementary material, to aid readability, and instead a stronger narrative summary of the documents is provided.

Another minor, semantic point but I would suggest you refer to the IPT theme as a conceptual label instead. Your IPT is actually your -if-then-leading to' statement, and it is this combined, that produces your IPT (which is more of an umbrella term for your 11 statements).

Discussion

Causal claims are supported, and the findings are situated in the broader evidence based, and linked back to the introduction and rationale. It would be good to hear from the team in terms of how this work will now be built on too.

Overall, this is a very strong paper, which is well written and clear, and I have only a few minor revisions to suggest, which I hope are helpful.

VERSION 1 - AUTHOR RESPONSE

**Reviewer 1 Miss Georgette Eaton, University of Oxford**	
Areas for strengthening<br>- The manuscript would benefit from greater clarity around mechanisms. In several places, mechanisms appear descriptive rather than generative, and some terms read more like organisational features (for example, leadership or teamwork) than underlying causal mechanisms such as reasoning, responses, or resources.	Thank you for raising this, we can see that as we were exploring this research question at the organisational level, there are several organisational features that did come through our developed initial programme theories. Our next phase of this work, 'testing' these IPTs will either expand, refute or refine these further, to develop more detailed causal mechanisms. And importantly, our planned future work, empirical research via a realist evaluation, will garner richer causal insights within contemporary maternity care delivery, than of that generated at this phase of the review.
- The distinction between context and mechanism is sometimes blurred, particularly where organisational features appear to be used in both domains.	We appreciate this point and found we aligned with the wider realist literature, that contexts and mechanisms may overlap depending on the situation. We address this issue in the next phase of the review.
- The search process needs to be reported more transparently. Greater detail is needed on how searches evolved and how inclusion and exclusion decisions were made.	We have added much more detail on page 7 and have included a page in Supplementary File 1 to show our iterative searches evolved and decisions were made.
-The exclusion of mechanisms at policy or system level is potentially problematic for realist work, as such mechanisms often shape organisational outcomes and may be highly relevant to the review question.	This is such a great point and one we debated at length! Through these lengthy discussions and with realist review methodologist expert input, we felt having a boundary for this work was important. Partly to retain international relevance of this phase of the review where the system/policy levels would differ. And partly, to ensure this review was achievable within the funded timeframe. However, it is such an important point, we have added it as a limitation on page 27-28.
- The decision to exclude CMOs that did not relate to all four outcome domains may have constrained theory development and removed potentially important explanatory material.	We appreciate this point and have now added it as a limitation for greater acknowledgement of this point – please see page 27-28.
- The analogue cut-and-group process is acceptable, but it needs to be described in more detail to allow readers to follow the analytic logic.	Thank you, we have added more detail on page 8-9.
- The role of stakeholders in the refinement process would benefit from greater	We have stated the stakeholders were involved with refining the IPTs – see page 9

clarification. It is not always clear whether they were validating, refining, or generating theory, or how differing views were handled.	where we solicited feedback on the IPTs to develop them further. We also provided a Supplementary File (3) showing the changes based on their feedback for the final IPTs.
- The manuscript would be stronger if it moved further beyond description and offered more explanatory synthesis, including patterns across studies, tensions or contradictions, and comparison of contexts where mechanisms appeared to work differently.	Thank you for this point, as the purpose of this manuscript is to only present the initial programme theories as developed from the initial literature search, we feel your point will be addressed in our next paper which was to 'test' these initial programme theories with the literature which allows for these aspects. We also recognise that empirical work via a realist evaluation would also generate a richer explanatory synthesis within the contemporary maternity care context.
**Reviewer: 2 Dr. Erica Gadsby, University of Stirling**	
Abstract says this paper reports the first phase of a review. But it doesn't explain the link to the next phase.	Thank you, we have re-written the abstract as per editorial feedback and have incorporated this point too.
P26 line 48-49 says that next phases is to 'test' the IPTs with the wider literature – interesting to consider how that will be done, given the complexity of the IPTs developed. Is testing with more literature the most logical next step? What might that look like?	Thank you for this point, it is expected as part of a realist synthesis that the IPTs be tested with the literature, but we do take your point and have included a sentence on p27 to reflect that empirical research via realist evaluation methodology should also be considered (and is something we hope to do ourselves).
Strengths and limitations: 'the approach is methodologically complex making them challenging to conduct' – wording needs amending.	We have reworded this sentence (see p27).
Some of the references seem wrongly attributed. For example, refs 22 and 44 are both used to support the explanation of what a realist review is (for). But neither of these refs relate to realist methodology – one is a qualitative systematic review, one is 'England N Maternity Transformation Programme'.	Thank you for spotting this, there appeared to be a glitch in our referencing software RefWorks when we used a certain style, which should now be corrected.
P10 line 8 – mentions papers were excluded for low methodological quality, but this isn't normally a criteria in a realist review. And it doesn't sit with the main criteria of richness and relevance. How was methodological quality assessed, and was a consistently reviewed across all documents?	Thank you for flagging this, we followed Prof Andrew Booth et al's approach of the three 'Rs' richness, relevance and rigour (methodological quality of a research study). But we accidentally omitted this detail in the screening and selection sub-section, so have now added this in on p8, which should then align appropriately with the results section.
P10 lines 18-33 – summary of included papers appears twice, at beginning and end of paragraph, but with small differences.	Thank you for spotting this! Has been amended

The lengthy supplementary docs were very difficult to engage with/interpret in the format presented.	Apologies that this was difficult to follow.
**Reviewer: 3 Dr. Ruth Abrams, University of Surrey**	
Intro: A minor point is that you mention drawing on international literature for this study. However I did wonder at this point whether this meant all countries including low to middle income, who have very different circumstances surrounding maternity care. Could the authors clarify in the introduction, which international literature they mean/ any boundaries around this up front?	Thank you for raising this, it is an important point – we have added some information to highlight we meant high-income countries on p.4.
However, can the rationale for only describing mechanisms be more clearly articulated in the paper. It is not typical to see mechanisms discussed in isolation, as part of the realist approach, largely because they do not operate in isolation, being instead triggered by their relational context.	Apologies, I think that was clunky/poor phrasing and we have inadvertently used the word mechanisms as a proxy for the whole IPT. We see that this has been confusing so have made small amendments throughout to enhance clarity and updated the title of the paper to convey this. Please see p5, p6 and p26.
Furthermore, when it comes to your findings- you articulate the statements in full so it feels like you have done yourselves a slight disservice by saying you've only got mechanisms, when actually you've done a lot more work- you have an entire IPT comprising 11 if-then-leading to statements. I'd strongly suggest framing your work in this way, to reflect it's comprehensiveness.	Thank you – this is very helpful and hopefully the amends above also address this comment.
Page 7, line 36 there is a typo here. Page 9, line 13, also a typo.	Thank you for spotting these, these have been amended.
Methods: My only concern with this section, which is repeated throughout as well is that you state you are using the heuristic, if-then-leading to. Typically in realist approaches, it is if-then-because, with the 'if' corresponding to context, then 'then' corresponding to the outcome, and the 'because' corresponding to the mechanism. Here you are using your 'then' to describe the mechanism, it would appear. I think this needs to be made explicit.	This is a great point, and we acknowledge there are different approaches within the realist field. We have added a sentence for clarity on p.8 to explicitly state what word corresponds to which part of the CMO configuration.
Findings: There are sentences in the study characteristics paragraph that are repeated.	Thank you for spotting this! Has been amended.
Suggest the table of document characteristics is moved to your supplementary material, to aid readability, and instead a stronger narrative summary of the documents is provided.	Thank you, we have opted to keep this study characteristics table as it is to help readers with less familiarity of realist reviews and it would typically be expected.
Another minor, semantic point but I would suggest you refer to the IPT theme as a	This is a great idea, thank you – we have changed this.

conceptual label instead. Your IPT is actually your -if-then-leading to' statement, and it is this combined, that produces your IPT (which is more of an umbrella term for your 11 statements).	
Causal claims are supported, and the findings are situated in the broader evidence based, and linked back to the introduction and rationale. It would be good to hear from the team in terms of how this work will now be built on too.	In addition to our sentence on p27 stating our next phase will be the testing phase as per realist synthesis, we have added a sentence about future work including realist evaluation to further develop our understanding within contemporary maternity services. This is an inference to what we hope will be our next steps, funding allowing!

VERSION 2 - REVIEW

Reviewer 2

Name Gadsby, Erica

Affiliation University of Stirling

Date 31-Jul-2026

COI

The changes made to the manuscript in this revised submission have addressed the reviewers comments and have strengthened the paper overall. I would consider this is valuable contribution to the literature and have no further suggestions.

Reviewer 3

Name Abrams, Ruth

Affiliation University of Surrey, School of Health Sciences, Faculty of Health and Medical Sciences

Date 06-Aug-2026

COI

Thank you to the authors for taking the time to consider the feedback. This was probably a particularly tricky task given you have three researchers, all very experienced in undertaking realist reviews themselves! The responses to reviewers are well considered, and where comments have been pushed back on, this has been done well. There are however a few points below to address, which I hope are helpful to consider:

1. Contexts as plural: Should the new title include context as plural, as oppose to singular? This comment also applies to the start of the discussion section too. You haven't got just one context. You have one setting (i.e. maternity care), but this is different.

2. Positioning this study: In the searches section, authors state that: "our search strategy sought to identify 10-12 candidate papers that were sufficiently rich, diverse, and relevant to our focus…". This therefore isn't a full realist review, but more of a scoping activity, to, as stated identify the candidate papers, to develop the IPT. Then you include 17 papers overall. With this extra detail, it now feels unclear what this step actually is- a scoping activity for candidate papers, or an extensive/ exhaustive realist review (which, if you are only developing an IPT, it then isn't a full review, because a full realist review would start with an IPT, and end with a final PT. There's nothing wrong with developing an IPT from a rapid review of literature, or a scoping activity designed specifically to generate an IPT but I'm not sure this can be referred to as a realist review. Whilst this might sound major, I don't think it is. You already clearly say that this is the first stage of a full review ("this paper reports on the first phase, in which 11 IPTs were developed from an extensive international evidence-base together with stakeholder input.")- but what needs to happen is that this is reported consistently throughout- i.e. you are not doing a realist review, you are creating an IPT. This is articulated well in the conclusion, but in the revisions it has become confused in the main body.

3. Table 1 content: Reviewing this now, with the fully articulated understanding of your heuristic (i.e. if= context; then =mechanisms; leading to= outcomes), I think you need to revisit some of your outcomes. Specifically, I'm talking about number 1, "LEADING TO a responsive, transparent, constructive, supportive mechanism for quality improvement…"- if it's a mechanism then it's not an outcome. Also, number 6, operational fitness sounds more like a mechanism. Finally, number 11, which appears to say a very similar, if not the same thing in both outcomes and could do with better synthesis into one outcome: "LEADING TO improving the quality, safety and inclusiveness of services for these groups LEADING TO staff and service users from the Global Majority communities feeling and being safer and having better experiences, improved staff work experience." The point from reviewer 1 about the blurring of boundaries between contexts and mechanisms I feel still stands. Whilst you say this overlaps in the literature, it is our job as researchers to untangle this. Mechanisms will rarely be located directly within the literature, because they are inferred, and generated over patterns across all 17 papers. I do think, given you are being encouraged to re-look at outcomes, it would be worth trying to tighten up all statements. Doing this now will make it much easier when you come to 'test' them in an evaluation, because you already have conceptual clarity. It doesn't matter if they are 'wrong'- you can note that as a finding in the empirical evidence, in due course.

I hope these three, relatively minor points are helpful in the next revision.

VERSION 2 - AUTHOR RESPONSE

**Reviewer: 3 Dr. Ruth Abrams**	
Contexts as plural: Should the new title include context as plural, as oppose to singular? This comment also applies to the start of the discussion section too. You haven't got just one context. You have one setting (i.e. maternity care), but this is different.	Thank you for pointing this out – it has been amended throughout.
Positioning this study: In the searches section, authors state that: "our search strategy sought to identify 10-12 candidate papers that were sufficiently rich, diverse, and relevant to our focus…". This therefore isn't a full realist review, but more of a scoping activity, to, as stated identify the candidate papers, to develop the IPT. Then you include 17 papers overall. With this extra detail, it now feels unclear what this step actually is- a scoping activity for candidate papers, or an extensive/ exhaustive realist review (which, if you are only developing an IPT, it then isn't a full review, because a full realist review would start with an IPT, and end with a final PT. There's nothing wrong with developing an IPT from a rapid review of literature, or a scoping activity designed specifically to generate an IPT but I'm not sure this can be referred to as a realist review. Whilst this might sound major, I don't think it is. You already clearly say that this is the first stage of a full review ("this paper reports on the first phase, in which 11 IPTs were developed from an extensive international evidence-base together with stakeholder input.")- but what needs to happen is that this is reported consistently throughout- i.e. you are not doing a realist review, you are creating an IPT. This is articulated well in the conclusion, but in the revisions it has become confused in the main body.	Thank you for raising this point, we have revised the title again to make this a clear signal to the reader that this is Phase 1 of a realist review: *'Understanding the contexts and mechanisms that drive high-quality maternity care outcomes within organisations: Generating 11 Initial Programme Theories as Phase 1 of a realist review.'*<br>We have further identified that this is Phase 1 of a realist review on pages: 1 (abstract), 4, 13 and 15.
Table 1 content: Reviewing this now, with the fully articulated understanding of your heuristic (i.e. if= context; then =mechanisms; leading to= outcomes), I think you need to revisit some of your outcomes. Specifically, I'm talking about number 1, "LEADING TO a responsive, transparent, constructive, supportive mechanism for quality improvement…"- if it's a mechanism then it's not an outcome. Also, number 6, operational	Thank you so much for raising these, it has been really helpful to have another pair of eyes on these IPTs, particularly with your expertise and we agree with these points. We have made various amends throughout the IPTs, tweaking and tightening up where needed, please see page 11.

fitness sounds more like a mechanism.<br>Finally, number 11, which appears to say a very similar, if not the same thing in both outcomes and could do with better synthesis into one outcome: "LEADING TO improving the quality, safety and inclusiveness of services for these groups LEADING TO staff and service users from the Global Majority communities feeling and being safer and having better experiences, improved staff work experience."<br>The point from reviewer 1 about the blurring of boundaries between contexts and mechanisms I feel still stands. Whilst you say this overlaps in the literature, it is our job as researchers to untangle this. Mechanisms will rarely be located directly within the literature, because they are inferred, and generated over patterns across all 17 papers. I do think, given you are being encouraged to re-look at outcomes, it would be worth trying to tighten up all statements. Doing this now will make it much easier when you come to 'test' them in an evaluation, because you already have conceptual clarity. It doesn't matter if they are 'wrong'- you can note that as a finding in the empirical evidence, in due course.<br>I hope these three, relatively minor points are helpful in the next revision.	

VERSION 3 - REVIEW

Reviewer 3

Name Abrams, Ruth

Affiliation University of Surrey, School of Health Sciences, Faculty of Health and Medical Sciences

Date 13-Aug-2026

COI

Dear Authors,

Thank you for again taking the time to address the feedback. Of course, an academic paper is never finished, and particularly when developing IPTs/ CMOcs- this could go on forever, but from a final review, I think all major points have been addressed. There is a legacy sentence in the conclusion from past iterations which doesn't quite match how the paper

has evolved, but I leave with you and the editors to address this in the proofing ("This paper reported on the first phase of a realist review exploring the mechanisms of high-quality maternity at the organisational level of service delivery") (i.e. not only mechanisms, as we've explored).

I look forward to seeing how this work progresses.

Thank you for the opportunity to review this manuscript.
