# Supplementary material for: Understanding the contexts and mechanisms that drive high-quality maternity care outcomes within organisations: generating 11 initial programme theories as phase 1 of a realist review
Source: BMJ Open. 2026 Aug 31;16(8):e119351. doi: 10.1136/bmjopen-2026-119351 (PMC13536141; doi:10.1136/bmjopen-2026-119351)
Supplement: Supplementary file 3 [file bmjopen-16-8-s003.docx]

# IPT Vignettes explored in the stakeholder meetings

## Theme 1: A listening, hearing, learning and acting culture: responding to staff and service user insights

| **IPT** | **Discussion during stakeholder event / Feedback given by stakeholders:** | **New IPT** |
| --- | --- | --- |
| IF organisations embed and endorse a systematic approach (e.g. investment in the required time and resources) to a listening, hearing, learning and acting culture* including data intelligence gathering inclusive of service users and staff alike THEN staff are more likely to implement a shared sense of ownership and responsibility for continual service improvements LEADING TO a responsive, transparent, constructive, supportive mechanism for quality improvement LEADING TO sustained high-quality care that is adapted to the population and individual needs in which staff and women feel respected and heard, particularly in case of adverse events  * Meaning: to listen and act on what is heard, troubleshooting, pre-empting adverse events. | - Dream Scenario, incredibly rare ✔ - Resonates with the group as a scenario when things are working well. ✔ - Reliant on individuals – organisation doesn’t facilitate this. ✔ (we mean an organisation carrying the collective responsibility to enable their staff to do this). A culture that is embedded and reinforced as the processes and systems are put in place. - Protected time needed to improve – time is main barrier ✔ - Ways of working another barrier – Quality and Governance/Audit team are separate and receiving feedback from different points in the system – no central system linking back = everyone working in silo. Need more co-production, everyone working together (with) not completing work on ✔ changes made - Resources needed = leadership and organisation = upskill staff in improvement work = best ideas come from shop floor ✔ - Need to be accountable to the communities = co-developed this together, whilst continually reviewing and developing = the quality improvement model. ✔ (added) - Who are Healthcare Professionals in question? ✔ (acknowledged) - Different cultures within diff teams and departments, Band 5s not included. ✔ - Don't use phrase journey = use experiences ✔ - Don't use making us feel = use ensure we are listened to. ✔ - There's a need here too for staff to be empowered to make changes themselves - quick wins! ✔   Change vignette?   - Service users should be at the centre of this theme, not healthcare professionals ✔ Final four will have vignettes from different stakeholders' points of views | IF organisations embed and endorse a central and systematic approach (e.g. investment in the required time and resources, central data intelligence gathering and sharing) to a listening, hearing, learning and acting culture* inclusive of service users and staff alike THEN staff are more likely to implement a shared sense of ownership and responsibility for continual service improvements LEADING TO a responsive, transparent, constructive, supportive mechanism for quality improvement LEADING TO sustained high-quality care that is adapted to the population and individual needs in which staff and women feel respected and heard, particularly in case of adverse events  * Meaning: to listen and act on what is heard, troubleshooting, pre-empting adverse events and disseminating lessons learned. Fostering robust accountability to the local community. |

## Theme 2: The organisation of maternity care is centred on women’s values and individualised/personalised needs by resourcing staff effectively

| **IPT** | **Discussion during stakeholder event / Feedback given by stakeholders:** | **New IPT** |
| --- | --- | --- |
| IF maternity services organise care centred on women’s values, where care is coordinated by a named professional and all staff have the appropriate support and resources* to provide relational care and maternal choice** across the spectrum THEN service users are able to participate in informed-decision making in a meaningful way while staff feel safe and able to discuss and justify personalised care plans (e.g. care that is ‘out of guidance’) LEADING TO higher quality care for all women, better outcomes and experiences for women and staff and a better use of resources.  * All maternity staff are enabled to develop meaningful and trusting relationships with women, provide evidence-based information clearly and impartially, listen closely to questions, preferences and decisions communicated by maternity service users, and respond to them in a clear and impartial way while mediating full scope safety & this care is led and coordinating by a lead care provider.  ** maternal choice is in place, both for the support of physiological processes, and for interventions where they are needed or wanted. | - Very happy with this theme ✔ - Title – is it centred around women’s values? Or Choices/autonomy/needs/wants/personalisation/women’s rights ✔ - Encompasses other care but not postnatal care = need to include/title, not maternity ✔ - My care during pregnancy and the postnatal period ✔ - Needs to discuss relationship within multi-disciplinary team = effective communication ✔ - Range of options – what is important to you as individuals – most accessible format to deliver this information? ✔ - Also, this theme makes staff feel they’re providing good holistic care ✔ - Need to not make women repeat stories – central system needed to ensure healthcare professionals understand medical history, needs, psychological needs) ✔ - Don't use pregnancy journey – use pregnancy experience to highlight our attention should be on providing a positive experience. ✔ - Valid and informed consent needed = can change mind at different point. ✔ - Time/staff/operable digital staff needed to understand all parts of care = good decision making. Training needed = some staff already do this well ✔ - Every woman needs all options available to them = staff need to be enabled and safe to do this. Are staff equipped and safe to do this? ✔ - Leadership and guidelines needed to facilitate this. ✔ - Right processes need to be in place to escalate things when needed. ✔ (not relevant to this IPT) - Don’t use good hands = safe caring hands ✔ - Need to do more than listen = take appropriate action as well. ✔ - Use of BRAIN tool to ensure women and clinicians are working together and both being listened to. ✔ (Is a ‘how’ or tool that can support all of this) | IF maternity services organise and invest in high-value care from the perspectives of those using the services*, where care is coordinated by a named professional who works within an aligned multi-disciplinary team and all staff have the appropriate support and resources** to provide relational care and maternal choice*** across the spectrum THEN service users are able to participate in informed-decision making in a meaningful way while staff feel safe and able to discuss and justify personalised care plans (e.g. care that is ‘out of guidance’) LEADING TO higher quality care for all women, seamless pathways, better outcomes and experiences for women and staff and a better use of resources.  * Evidence-based and personalised, high value maternity care across the continuum and across all settings (which are appropriately and equitably resourced, ringfenced, and accessible* to all women)  ** All maternity staff are enabled to develop meaningful and trusting relationships with women, provide accessible evidence-based information clearly and impartially, listen closely to questions, preferences and decisions communicated by maternity service users, and respond to them in a clear and impartial way while mediating full scope safety & this care is led and coordinated by a lead care provider.  *** maternal choice is in place, both for the support of physiological processes, and for interventions where they are needed or wanted. |

## Theme 3: High-quality maternity adopts a broader definition of safety (beyond just physical safety)

| **IPT** | **Discussion during stakeholder event / Feedback given by stakeholders:** | **New IPT** |
| --- | --- | --- |
| IF maternity organisations, leadership and staff adopt a perspective of safety that includes both technical/clinical safety and psychological, emotional and cultural safety as defined by all stakeholders involved in maternity care which is embedded throughout all governance structures and resourcing, THEN all stakeholders adopt and champion a broad perspective on safety, creating a clinically, emotionally, culturally and psychologically safe environment for all LEADING TO all four outcome indicators. | - Statement was found to be great by the stakeholders. If we would be doing this, we would not have the issues that currently exist. ✔ - Holistic notion of safety and importance of a midwife that cares and which you trust. ✔ - Negative experiences or trauma at birth usually to do with the lack of emotional support and lack of relational care rather than with what happened medically. ✔ - Need to care for the carer. Centering the emotional well-being of staff to enable them to be compassionate. ✔ - Triangulation very important, Trust boards etc should not just be focussed on outcomes but on service user feedback, team culture (Score surveys etc), staffing levels, resources, time, flexibility etc.  So very important. A happy team (workforce) is very important to enable us to give excellent care. ✔ - psychological harm is often difficult to measure as it is often more apparent many weeks after birth, so we need to be better at gathering information/data. ✔ - Effective measurements of psychological as well as physical safety ✔ | IF maternity organisations, leadership and staff adopt a perspective of safety that includes both technical/clinical safety and psychological, emotional and cultural safety as defined by all stakeholders involved in maternity care which is embedded throughout all governance structures intelligent data gathering*, and resourcing, THEN all stakeholders adopt and champion a broad perspective on safety, creating a clinically, emotionally, culturally and psychologically safe environment for all LEADING TO all four outcome indicators.  *Triangulation of data gathering (outcomes and service user feedback (in the short and long-term), staffing levels, resources, time, flexibility, etc.) |

## Theme 4: Visible and supportive leadership enhancing the workplace and safety culture

| **IPT** | **Discussion during stakeholder event / Feedback given by stakeholders:** | **New IPT** |
| --- | --- | --- |
| IF maternity leaders manage hierarchies flexibly and strategically* while being highly visible on the shop floor, supporting and setting clear standards for what is considered appropriate actions and decisions, explaining how and why, allowing colleagues to develop their skills through reflection-in-action THEN staff feel supported and enabled to develop their skills in united, passionate, coordinated and stable teams LEADING TO actions taken that are based on the requirements of the situation rather than on hierarchies protecting organisational interests, LEADING TO better outcomes and experiences for women and staff, and a better use of resources.  * Achieving a balance between supporting the learning and allowing staff to make autonomous decisions.  ** hierarchies can be mobilised when needed to clarify social norms and behavioural standards but can be muted where not required | - Flexibility means that leader's roll up their sleeves and do the day job when this is needed, leaders understand the day job because they do it. Having leadership at all levels and focus on knowledge and skills that leaders need to develop. ✔ - Reflection (not only on what goes wrong but also on what goes right, positive feedback). ✔ - Safe escalation routes. ✔ - it's also about breaking down hierarchies which is quite counter-cultural within the NHS. ✔ - Leadership comes from all 'levels' ✔ - How to be a very safe maternity unit: An ethnographic study – ScienceDirect – breaking down hierarchical barriers. ✔ - Who decides what ‘safe care’ means? Lived experience, culture, and values women. ✔ - Maternity care continues to be associated with avoidable harm that can result in serious disability and profound anguish for women, their children, and families. ✔ - Importance of visible leadership and to enable staff to thrive in their role, to develop expertise in the field they are interested in and this in an equitable way. ✔ - Professional autonomy. ✔ - Multidisciplinary team cohesion by spending time together in one break room? - Psychological safety – No voice is unheard. A culture in which everyone feels safe to speak up. ✔ - Moving away from tick box exercises  holistic approach to care. ✔ - Even though some units might be seen as high performing in view of certain indicators, sometimes there is still big cultural problems ongoing under the surface (e.g. ‘them and us culture’ between AMU and labour ward). ✔ | IF knowledgeable and skilled maternity leaders manage hierarchies flexibly and strategically*, role model, while being highly visible on the shop floor, supporting and setting clear standards for what is considered appropriate actions and decisions (including safe escalation routes), explaining how and why, allowing colleagues to develop their skills through reflection-in-action*** THEN staff feel supported and enabled to work autonomously, to develop their skills within the full scope of their practice in united, passionate, coordinated and stable teams LEADING TO actions taken that are based on the requirements of the situation rather than on hierarchies protecting organisational interests, LEADING TO better outcomes and experiences for women and staff, and a better use of resources.  * Achieving a balance between supporting the learning and allowing staff to make autonomous decisions.  ** hierarchies can be mobilised when needed to clarify social norms and behavioural standards but can be muted where not required  *** aligned with psychological safety and attendance to the three core needs of autonomy, belonging and contribution |

## Theme 5: All maternity care systems, processes, procedures and care are grounded in evidence-based frameworks

| **IPT** | **Discussion during stakeholder event / Feedback given by stakeholders:** | **New IPT** |
| --- | --- | --- |
| IF the organisation employs ‘best practice’ evidence-based frameworks to support all systems and processes including governance (e.g., guideline development, guideline developers being at the forefront of guideline methodology, quality improvement), THEN an evidence-informed inclusive, collaborative, and participative approach can flourish encompassing full-scope safety, effectiveness, and person-centredness LEADING TO the adoption, delivery and sustainability of evidence-based person-centred care in which providers can make the right decisions at the right time based on women’s choices and needs. | - Important to consider who decides what ‘evidence-based’ is and who has done the research. Who benefits from the evidence? ✔ - Evidence-based practice needs to consider the individual women and their needs. A holistic view is necessary and the importance of culturally appropriate care. ✔ - The evidence-based guidelines need to constantly evolve and be updated regarding the latest research that is done. So, practice also continuously evolves (not getting stuck in the ‘we have always done it this way’. ✔ - The team needs to be on the same page and give all women information in line with the current guidelines and evidence available (not anecdotal information from their perspective or point of view). Consistency is needed. ✔ - Leadership and especially labour ward coordinators need to be role models in always being up to date with the latest evidence and knowing the guidelines inside out so the team can work in a cohesive way. ✔ (see leadership IPT) - Culture is important but also flattening hierarchies so that also the more junior staff has a say in the development and practice of evidence-based practice. ✔ - training plays a role in achieving optimal performance ✔ (optimal clinical performance? - Making it easy for people to do the right thing! ✔ | IF the organisation employs ongoing ‘best practice’*evidence-based frameworks to support all systems and processes including governance** led by appropriately skilled professionals, THEN an evidence-informed inclusive, collaborative, and participative approach can flourish encompassing full-scope safety, effectiveness, and person-centredness LEADING TO the adoption, delivery and sustainability of evidence-based person-centred care in which providers can make the right decisions at the right time based on women’s choices and needs.  *‘Best Practice’: Evidence-based practice which exists of the triad of woman’s needs, best available clinical evidence and clinical expertise.  **e.g. for guideline development, the team should have appropriate research (systematic review) skills. (e.g. a fundamental valuing and reinforcement that all these things should be done by people with the appropriate knowledge and skills). |

## Theme 6: The system & workplace environment supports optimal clinical performance

| **IPT** | **Discussion during stakeholder event / Feedback given by stakeholders:** | **New IPT** |
| --- | --- | --- |
| IF organisations design, implement and continually assess their working environments including systems, processes, structures, equipment, IT are optimised aligned with human factors/ergonomics and Safety II principles and deploy intelligent data gathering (soft and hard) to manage system risk THEN the safety of the ‘system’ is enhanced/optimised with features of increased flexibility, preparedness and responsiveness to actual events/needs of the service (rather than reactive) LEADING TO decreased cognitive burden for staff and enabling staff to work to their full scope of practice (e.g., using clinical skills not administrator skills) LEADING TO operational ‘fitness’ within an organisation improving outcomes and experiences of care/workplace. | - Currently, there is many issues with IT and systems. ✔ - Reduction of paperwork or having to duplicate notes on paper and different online systems. ✔ - Medicines and equipment need to be available for all staff (not one drug key that is with the coordinator but all staff able to enter the drugs cupboard). ✔ great example - Flattened hierarchies: All staff having a say around what works well and what does not work, what is missing and should be put in place. Culture in which leadership listens, hears, acts. ✔ - Technology and other equipment need to consider the maintenance of positive relationships with women and birthing people. Ensuring that healthcare providers can have a good body language, etc. Governance or technology that frees up more time to be ‘with women’ and to care. 🡪 Rival theory? ✔ great example - Safe staffing for whom breaks are prioritised. ✔ - Health and safety ✔ | IF organisations design, implement and continually assess / review their working environments including systems, processes, structures, equipment, IT and facilities are optimised aligned with human factors/ergonomics* and Safety II principles and deploy intelligent data gathering (soft and hard) to manage system risk THEN the safety of the ‘system’ is enhanced/optimised with features of increased flexibility, preparedness and responsiveness to actual events/needs of the service (rather than reactive) LEADING TO increased staff wellbeing, decreased cognitive burdens and enabling staff to work to their full scope of practice (e.g., using clinical skills not administrator skills) LEADING TO operational ‘fitness’ within an organisation and optimal care delivery where staff are enabled to do the right thing consistently, better teamwork and job satisfaction, retention of the workforce and high-quality care provision.    * Psychological safety and attendance to the three core needs of autonomy, belonging and contribution  (e.g. examples of basic facilities and staff support: places and time to rest and sleep, access to nutritious food and drinks, the tools needed for staff to do their job (e.g. MDT training, (clinical) support, implement educational packages, respect, autonomy, consideration of work schedules based on realistic forecasting and demands that accounts for fatigue, supports safe shift swapping and enables breaks consistently and also providing easily accessible workplace tools to support clinical activity e.g., Wi-Fi, mobile desktops and evidence-based clinical resources, pathways and guidelines that help staff to do the right thing, a practical lay-out of the ward, etc.) |

## Theme 7: The organisation and its systems are adapted to staff needs

| **Old IPT** | **Feedback** | **New IPT** |
| --- | --- | --- |
| IF maternity care organisations ensure minimum standards for basic facilities and staff support* aligned with psychological safety and attendance to the three core needs of autonomy, belonging and contribution THEN this improves working conditions, optimises health and wellbeing for staff (as they feel valued, supported and empowered), LEADING TO optimal care delivery where staff are enabled to do the right thing consistently, better teamwork and job satisfaction, retention of the workforce and high-quality care provision.  * Examples of basic facilities and staff support: places and time to rest and sleep, access to nutritious food and drinks, the tools needed for staff to do their job (e.g. MDT training, (clinical) support, implement educational packages, respect, autonomy, consideration of work schedules based on realistic forecasting and demands that accounts for fatigue, supports safe shift swapping and enables breaks consistently and also providing easily accessible workplace tools to support clinical activity e.g., Wi-Fi, mobile desktops and evidence-based clinical resources, pathways and guidelines that help staff to do the right thing, a practical lay-out of the ward, etc.) | - Mixed feelings about the vignette: sounds like the perfect job / what people would want but not realistic, this should be basic and not up for discussion, this is not appealing to me or what I would want as a hcp, this is what some members of the multidisciplinary team get but others don’t. ✓  - It is only a small piece of the puzzle to provide great maternity care and not a priority. ✓  - Equity, psychological and cultural safety, parking, training, education, racism, blame culture, etc. ✓  - It is very difficult to measure a manageable workload (as acuity constantly changes) but it needs to be more manageable. ✓ (covered in other IPT)  - Ratio’s in maternity services are often too high and not really focused on much (1RM:16 PN women) ✓ (covered in other IPT) - The organisational culture is the context in which we care ✓ | IF maternity care organisations ensure minimum standards for basic facilities and staff support* for all aligned with psychological safety and attendance to the three core needs of autonomy, belonging and contribution THEN this improves working conditions, optimises health and wellbeing for staff (as they feel valued, supported and empowered), LEADING TO optimal care delivery where staff are enabled to do the right thing consistently, better teamwork and job satisfaction, retention of the workforce and high-quality care provision.  * Examples of basic facilities and staff support: places and time to rest and sleep, access to nutritious food and drinks, the tools needed for staff to do their job (e.g. parking, MDT training, (clinical) support, implement educational packages, respect, autonomy, cultural safety, consideration of work schedules based on realistic forecasting and demands that accounts for fatigue, supports safe shift swapping and enables breaks consistently and also providing easily accessible workplace tools to support clinical activity e.g., Wi-Fi, mobile desktops and evidence-based clinical resources, pathways and guidelines that help staff to do the right thing, a practical lay-out of the ward, etc.) |

## Theme 8: Leadership drives a high-quality service by attending to the triad of safety, service user experience and clinical effectiveness

| **Old IPT** | **Feedback** | **New IPT** |
| --- | --- | --- |
| IF maternity leaders ensure that all aspects of the quality triad – safety, patient experience / person-centredness and clinical effectiveness are in place by authentically involving service-users and acting on their views THEN this facilitates strong, compassionate, and courageous leadership and management enabling the MDT to work well across the system LEADING TO a culture of caring, choice, and a strong service-user voice. | - Focus on the public is important, in practice not quite nailed. Who we talk to doesn’t currently represent all service users – SU has a small voice. Policy and rules sound great but require more guidance on how to get this right.  ✓  - We are still in a space where the environment needs to be enabled to speak first before this can occur. ? unclear  - This IPT seems like a system led as opposed to community leading and being in control. Input from the community needs to be received well, listened to and acted upon – with accountability measures in place to ensure a back-and-forth discourse to achieve the desired outcomes. ✓  - Who defines what is optimal? ✓ (person-centredness, optimal for the individual)  - We need the staff to be engaged and service users to be diverse and reflective of the community we are serving. We also need to hear from student midwives and staff on lower bands. We also need to ensure we don’t discount the range of HCPs from this discussion as they see more families. Need to produce a more joined and equal-weighed team. ✓  - Friends and Family Test needs to be translated into usable data, even if it’s just an AI tool to analyze the data so it can be used in practice. ✓ (acting on SUs views)  - We need triangulation of who/what/how people engage with this IPT. Peer-to-peer engagement is needed. ✓ * | IF maternity leaders ensure that all aspects of the quality triad – safety, patient experience / person-centredness and clinical effectiveness are in place by authentically and proactively involving all staff and a wide range of service-users and acting on their views*; working with the local community and voluntary sector organisations THEN this facilitates strong, compassionate, and courageous leadership and management enabling the MDT to work well across the system LEADING TO a culture of caring, choice, and a strong service-user voice.  *e.g. intelligent data gathering and sharing is triangulated with all stakeholders to ensure accountability of senior teams |

## Theme 9: Responsiveness to ongoing workload and acuity assessments accounting for high-value care and the different workplace contexts

| **Old IPT** | **Feedback** | **New IPT** |
| --- | --- | --- |
| IF organisations implement and sustain a programme to review workload in their organisations that takes account of the specific context and work that is done in different areas of the service and the nature of high-value care from the perspectives of those using the service, THEN the service can be adequately and appropriately resourced across the board, teams are protected to provide their services while also tackling excessive work demands on staff LEADING TO sustainability in delivering safe, high-value, high-quality care and meeting core staff needs so service users receive high-quality, safe, and compassionate care. | - Very key 🡪 if systems and the acuity does not reflect actual activity, this is problematic. ✓ - The recognition that the current tool used is not the best is important. ✓  - An on-call system can be very psychologically draining and is not always financially desirable. ✓  - Having a pool of midwives available when there is shortages would be ideal but there is likely not enough financial resources nor midwives available to do this. ✓  - This vignette has a big impact on safety. ✓  - Safe staffing often purely focuses on numbers rather than skill mix and experience. ✓- From a service-users point of view, women and their families want to feel safe and expect that staff have the right skills and experience. ✓ | IF organisations implement and sustain a programme to review workload in their organisations that takes account of the specific context and work that is done in different areas of the service (with appropriate skill mix) and the nature of high-value care from the perspectives of those using the service, THEN the service can be adequately and appropriately resourced across the board, teams are protected to provide their services while also tackling excessive work demands on staff LEADING TO sustainability in delivering safe, high-value, high-quality care and meeting core staff needs so service users receive high-quality, safe, and compassionate care. |

## Theme 10: Resource management for meaningful choice provision and optimal care with seamless care pathways for all service user needs

| **Old IPT** | **Feedback** | **New IPT** |
| --- | --- | --- |
| IF organisations implement and sustain evidence-based and personalised, high value maternity care across the continuum and across all settings (which are appropriately and equitably resourced, ringfenced, and accessible* to all women) THEN seamless pathways exist for all women (e.g., both a ELCS AND homebirth pathway are available), meeting individual needs whilst addressing inequalities LEADING TO women and babies being offered the right care at the right time LEADING TO improved outcomes, improved wellbeing, optimal use of resources.  * Meaning: assurance of geographical, social, and economic access for all women. | - Best available evidence = not true or reflective of the current context. ✓ - ECV was not present as an option in a recent case, information shared with patient was copied and pasted from the RCOG website without one paragraph on ECV. Why don’t we at least signpost to proper materials. ✓  - Few women offered all four options. One survey showed 9% of 1500 women were offered all 4 = not the experience of majority of women ✓ - How is the evidence presented? Obstetricians unaware of entire evidence base offered. Women struggled to get Elective Caesarean Section, not available or presented to all women. Who decides what is the best evidence? Currently informed by HCPs individual experience. HCPs can often use abstracts as opposed to actual research. HCPs use only RCT instead of all evidence available; they need to ensure all data is used. ✓ (in another IPT) - Some women do not want all this information and feel overwhelmed and do not appreciate “I can’t make this decision for you”. ✓ (should come with personalised care) – responsive added - We need more time than just 10 minutes to facilitate this happening as well as continuity of care with effective communication between cross-working departments. ✓ (appropriately resourced) - Need local data to make informed choices as opposed to national data.✓ (in other IPT)  - Things aren't always presented to women for them to agree – decisions are made on their behalf. To achieve informed consent, we need support of staff to make this feasible and organisation support. Human Right’s element to this. ✓ (covered in this IPT). | IF organisations implement and sustain evidence-based, responsive, and personalised, high value maternity care across the continuum and across all settings (which are appropriately and equitably resourced, ringfenced, and accessible* to all women) THEN seamless pathways exist for all women (e.g., both a ELCS AND homebirth pathway are available), meeting individual needs whilst addressing inequalities LEADING TO women and babies being offered the right care at the right time LEADING TO improved outcomes, improved wellbeing, optimal use of resources.  * Meaning: assurance of geographical, social, and economic access for all women. |

## Theme 11: A culture of equitable care (underpins all other IPTs too)

| **Old IPT** | **Feedback** | **New IPT** |
| --- | --- | --- |
| IF the maternity service engages in meaningful ways with marginalised and minoritised groups, including socially disadvantaged and ethnic minoritised communities and commits to co-producing anti-racist policies and practices while providing cultural safety training for staff THEN the culture of the organisation and frontline staff perpetuate inclusive, respectful and ethical behaviours LEADING TO improving the quality, safety and inclusiveness of services for these groups LEADING TO staff and service users from the Global Majority communities feeling and being safer and having better experiences, improved staff work experience. | - IPT is the farthest away from reality as all of this is currently missing but most important to stakeholders. ✓  - How do you do anti-racism policies well – not too targeted or are there other pitfalls we are not aware of yet. ✓ (this hopefully will be uncovered in the next phases of the project, ‘how’)  - People do not feel listened to regarding Black and Global Majority groups' personalised care. ✓  - Lack of interpreter services, institutions have paid for it and it wasn’t translated well. Interpreter services huge priority. ✓  - A lot of women need interpretation services. The interpretation services are often via phone instead of face-to-face (which is a barrier at times and takes the fluency out of the consultation) and there is no continuity (some women ask for a female interpreter but often there is none available). This makes it impossible to provide what matters to women. ✓  - In some trusts they employ multilingual doula’s which could be the solution to continuity. ✓  -Respectful and dignified care not delivered in a responsive way ✓  - Cultural differences are not always recognised or respected. Being listened to be is a big element as well as making people feel safe to speak, merely asking questions to see if woman is ok is not enough as there are differences in health system practice or women may trust authority over themselves i.e. you said I am well so i must be well even if there is something else potentially going on with their bodies. ✓  - In predominantly white areas, additional support needed to facilitate safe and open relationships between white HCPs and Black and Global Majority groups as the HCPs will not have much practice building relationships with these demographics ✓ (we will address this in the next phase of the study).  - Leadership and work culture play a huge role in this. ✓  - A lot of work is currently based on the White British woman, with research and everything else based on the White Male, so we are a long way away from achieving this IPT = big gaps here to get better at this. Need to keep intersectionality at the forefront of this. Maternity is currently informed by the UK culture – how does this reflect in personalised care for Black and Global Majority Groups. - Women are coming with a lot of social issues (e.g. housing, not speaking English, etc.) which are things that should have been dealt with in community (should not be the responsibility of the maternity care organisation). ✓  - Important to join forces with services and other agencies in the community or to signpost to voluntary sector, NGO sector, organisations complementing NHS care (NHS Trusts that already do this are likely high performing ones). ✓  - There is often an assumption about health literacy. ✓  - Often the expertise and time of people of global majority backgrounds that help to write policy etc. are not valued. ✓ | IF the maternity service engages in meaningful ways with marginalised and minoritised groups, including socially disadvantaged and the global majority and commits to co-producing anti-racist policies and practices* while engaging local voluntary groups and the local community sector and providing cultural safety training for staff THEN the culture of the organisation and frontline staff perpetuate inclusive, respectful and ethical behaviours LEADING TO improving the quality, safety and inclusiveness of services for these groups LEADING TO staff and service users from the Global Majority communities feeling and being safer and having better experiences, improved staff work experience.  *e.g. culturally appropriate interpretation and/or doula services ensuring continuity. |
