# Supplementary material for: Understanding the contexts and mechanisms that drive high-quality maternity care outcomes within organisations: generating 11 initial programme theories as phase 1 of a realist review
Source: BMJ Open. 2026 Aug 31;16(8):e119351. doi: 10.1136/bmjopen-2026-119351 (PMC13536141; doi:10.1136/bmjopen-2026-119351)
Supplement: Supplementary file 4 [file bmjopen-16-8-s005.docx]

Table 1 Study characteristics

| Source/title | Publication type | Paper focus | Outcome indicator | Setting | Methods (overarching) | Data insights |
| --- | --- | --- | --- | --- | --- | --- |
| Crowe, C., & Manley, K. (2019)  Person-centred, safe and effective care in maternity services: the need for greater change towards best practice. | Systematic review | To identify learning from relevant inquiries into the  quality, safety and variation of maternity services, to develop understanding about what best practice looks like in maternity settings, and the relevant contextual factors important when implementing best practice in an NHS maternity service. | High rates of maternal wellbeing | Literature from the UK, England, Ireland, Switzerland, and Australia. | Five inquiries with relevance to maternity practice since 2013 (one local with national  recommendations, two national and two international), two subsequent national reviews and a further  17 key service publications over 10 years from the grey literature have been identified as sources of  data for analysis of best practice. | Three key themes were distilled: framing best practice in relation to the quality triad  of patient experience, safety and clinical effectiveness; the need to implement the lessons learned  from inquiries into quality and safety; and the importance of contextual factors including leadership,  learning and teamwork as enablers of best practice.  Implementing best practice and learning from quality of care inquiries are identified as key challenges when providing person-centred, safe and effective care mediated through contextual factors such as learning, leadership and teamwork. |
| De Jonge, A., Downe, S., et al. (2019)  Value based maternal and newborn care requires alignment of adequate resources with high value activities. | Commentary | To deliver value based maternal  and newborn care, a system-level shift is needed, ‘from fragmented care focused on identification and treatment of  pathology for the minority to skilled care for all’. | Optimal use of resources with judicious use of procedures | Papers from low, middle, and high-income countries. Public health systems. | A commentary including 29 references | To achieve value based maternal and newborn care, mechanisms are needed to ensure adequate resource allocation to high value maternity care activities that should be separate from the resource demands of acute maternity services. Funding arrangements should support, where wanted and needed, seamless movement of women and neonates between systems of care |
| De Leo, A., Bayes, S., et al. (2021)  Midwifery leaders’ views on the factors considered crucial to implementing evidence-based practice in clinical areas. | Primary qualitative research. | To explore midwifery leaders’ views on what information and support midwives require to lead practice change initiatives in clinical areas. | Overlapping category papers | Australia (public health sector) | The study formed part of a broader Participatory Action Research (PAR) project designed to improve the processes by which midwives implement evidence-based practice change in clinical settings. 8 midwives in leadership positions participated in a focus-group or face-to-face interview. | To lead practice change initiatives, midwives require a web-based resource that standardises the process of evidence implementation, while providing midwives with clear direction and the support needed to confidently champion for evidence base change in clinical areas. |
| Downe, S., Finlayson, K., & Fleming, A. (2010).  Creating a collaborative culture in maternity care. | Commentary | Presents current accounts of collaboration—or lack of it—in maternity care in the United Kingdom, United States, and Australia, examining tools designed to measure collaboration and teamwork within general health care contexts – to generate a set of characteristics for effective collaboration in maternity care. | Overlapping category papers | United Kingdom, United States, and Australia. | A commentary including 31 references. | - Authentic collaboration based on mutual trust and appreciation underpins success in both increasing maternal and fetal safety and normalising childbirth.  - The examination of a range of articles exploring practical and philosophical approaches to teamwork and collaboration in health care in general has resulted in a synthesis of possible characteristics for effective collaboration in maternity care in particular. |
| Kroll-Desrosiers, A. R., Crawford, S. L., (2016)  Improving pregnancy outcomes through maternity care coordination: a systematic review. | Systematic review of observational studies | To examine maternity care coordination (MCC) programs and their association with pregnancy outcomes. | Low rates of adverse outcomes | International | Thirty-three observational studies of MCC were included in this systematic review | Due to the heterogeneity of the studies, a meta-analysis could not be performed, However, results indicated maternity care coordination was associated with improvements to gestational weight and age (thus decreased neonatal morbidity), improved shared decision-making and MDT working, improved access and referrals to other agencies (social or mental health services). Limitations from the individual studies were note e.g., maternal postnatal depression were not included. |
| Liberati, E. G., Tarrant, C., et al. (2019)  How to be a very safe maternity unit: an ethnographic study. | Primary research | To characterise what makes Southmead safe, attending both to features of context and intervention to generate an in-depth understanding. | Low rates of adverse outcomes | Southmead Hospital (Bristol, UK) has a large maternity unit (approximately 6500 births per year) serving some of the most deprived areas of Bristol. | An ethnographic study involving observations (143 hours), semi-structured interviews (n=12), and focus-groups. | -The improvement logics of intervention and context should be understood as a unity, not a duality. - Safety, especially in acute care settings such as maternity care is an emergent property of complex systems: enduring high performance is likely to be dependent on reshaping the multiple, interacting features of systems (including specific interventions) in a way that enables the reproduction of desired outcomes. |
| McGowan et al., (2023)  Quality and reporting of large-scale improvement programmes: a review of maternity initiatives in the English NHS | Systematic scoping review | To identify large-scale improvement programmes focused on intrapartum care implemented in English National Health Service maternity services in the period 2010–2023, and to conduct a structured quality assessment. | Overlapping category papers | The English National Health Service maternity services. | Preferred Reporting Items for Systematic Reviews and Meta-Analyses extension for Scoping Reviews guidance were used to inform the design and reporting 1434 records via databases and other sources were identified. 14 major initiatives in English maternity services could not be quality assessed due to lack of a retrievable evaluation report. Quality assessment of the 15 improvement initiatives that could be included was carried out. | Poor transparency of reporting and weak or absent evaluation undermine large-scale improvement programmes by limiting learning and accountability. This review indicates important targets for improving quality in large-scale programmes. |
| Miller, S., Abalos, E., et al. (2016)  Beyond too little, too late and too much, too soon: a pathway towards evidence-based, respectful maternity care worldwide | A systematic review | To present the findings of a systematic review of evidence-based clinical practice guidelines for routine antenatal, intrapartum, and postnatal  care, categorising them as recommended, recommended only for clinical indications, and not recommended. Overall, to address the issues of ‘too little, too late and too much, too soon’. | Optimal use of resources with judicious use of procedures | Evidence-based guidelines from USA, Canada, UK, Germany, Argentina, Australia, the Basque Country, Brazil, Canada, Colombia, Ecuador, Italy, Japan, New Zealand, Norway, Scotland, Spain. (No guidelines developed by LICs met the criteria for inclusion). | 78 individual or groups of interventions recommended for use in routine antenatal (25), intrapartum (28), and postnatal (25) care in low-risk women attended at health facilities were extracted. | 37 individual or groups of interventions which guidelines recommended were not used in routine maternity care were identified.  19 recommended-against antenatal care interventions, ten concerned assessments and diagnostic and screening procedures of maternal and perinatal complications, seven were for prevention of specific pregnancy complications, and two were for organisation of antenatal care. Of 14 recommended-against interventions for intrapartum care, four interventions regarded assessment and monitoring of the progress of labour and maternal and fetal health, four were for pain relief, and six were for specific care in the different stages of labour. Four recommended-against interventions for postnatal care were also identified.  All of which identify key recommendations for future clinical guidelines to account for ‘too much too soon and too little too late’. |
| North East and North Cumbria Local Maternity and Neonatal System (2023)  Beyond too little, too late and too much, too soon: a pathway towards evidence-based, respectful maternity care worldwide | Report of a collaborative learning and improvement event | The aim of the event was to:  • Bring together colleagues from across the North East and North Cumbria who have been  involved in the recent Ockenden assurance visits  • Share themes and learning from the visits  • Enable each Trust to showcase areas of outstanding practice to support collaborative  learning and improvement across the NENC  • Chance for reflection and to identify solutions to the challenges we face  • To celebrate, listen and learn as a NENC maternity and neonatal community | High rates of staff wellbeing | 115 people attended the event, drawn from all areas including the 8 Provider  Trusts, MNVP Leads, LMNS Service User Voice Representatives, LMNS PMO & Clinical Leadership  team, NENC LMNS Board, NENC Maternity & Neonatal Alliance Board, NENC Integrated Care Board  and other stakeholders. | A report was drawn from the different presentations of the event. | The Post Ockenden Assurance Visits (2023) Learning & Improvement Event enabled Trusts to show case several different examples of outstanding practice that were seen as exemplars throughout the  visits and to also identify our system wide 'tricky issues' and to consider how we might start to  address those issues together. |
| Renfrew, M. (2024)  Enabling Safe Quality Midwifery Services and Care in Northern Ireland | Primary research, national investigation of the Northern Ireland Maternity Services | This report is an examination of midwifery and the wider maternity care and services  across NI. It arose firstly from a Coroner’s inquest into a baby’s death, with concerns  about safety in a Freestanding MLU.  The aim of the work was to identify the key conditions for safe, quality midwifery and  wider maternity services in all settings in NI, to ensure that safe, equitable, respectful,  compassionate, and evidence-informed midwifery care is available for all women and  newborn infants, wherever and whenever care takes place. | High rates of maternal wellbeing | Fifteen positive examples of midwifery services used were from NI (n=11), Scotland (n=1), England (n=1), Wales (n=1), and Spain (n=1). | The methods were designed to ensure the involvement and participation of women and families, advocacy groups and charities, midwives and midwifery students, and interdisciplinary professional colleagues  throughout. The perspectives of managers, leaders, and senior decision-makers working  in service provision and in the organisations responsible for service commissioning and  governance were also sought. 107 service users, 159 midwifery staff, 30 interdisciplinary colleagues, and 80 participants in a cross-sectional workshop were included. 34 meetings were held with senior decision makers and representatives of health and social care agencies.  - Eight rapid efficient evidence reviews were carried out. - Fifteen positive examples of midwifery services were used to identify characteristics of successful services (11 from across NI, one each from Scotland, England, Wales, and Spain. - Responses to questionnaires were received from all heads of midwifery, all HSC Trust Boards, and service and education commissioners.  - A systematic analysis drawing together and learn from all the findings. | Among extensive findings and reporting, 32 recommendations were developed taking a whole system approach. |
| Rönnerhag, M., Severinsson, E., et al. (2018)  Qualitative study of women's experiences of safe childbirth in maternity care. | Primary qualitative research | The aim of this study was to explore the meaning of safety as a process phenomenon by outlining women's positive and negative experiences of safety in childbirth | High rates of maternal wellbeing | The participants consisted of women recruited at one regional hospital in Sweden and invited to participate in the study during a follow-up visit to the maternity clinic. | Descriptive exploratory design including 16 interviews. Qualitative content analysis was used. | One main theme emerged: safe childbirth through involvement and guidance, based on four subthemes. The characteristics of women's experiences of safe childbirth included the need to be informed and involved by sharing and receiving trustworthy information. Women's experiences of unsafe childbirth included lack of meaningful and trustworthy information that resulted in feelings of being misled or lulled into a false sense of security. Not being involved evoked feelings of being ignored. In conclusion, this study highlights issues of importance for safe maternity care. |
| Russell, K. (2018)  Factors that support change in the delivery of midwifery led care in hospital settings. A review of current literature. | A narrative review. | To identify factors that support change in the delivery of the midwifery led care in hospital settings. | Overlapping category papers | Literature published in the UK, Sweden, Canada, and Australia | A narrative review  Eight high quality papers were selected for review. Papers focused on improving the delivery of midwife led care in hospital midwifery units, labour and postnatal wards. | The findings demonstrated the importance of social support and clinical leadership in bringing about subtle changes in hospital-based midwifery led care. Ultimately improved understanding of the factors that support the delivery of the midwifery led care in hospital settings may improve women’s choice and highlight the role of the midwife as the practitioner of normal childbirth. |
| The Healthcare Improvement Studies Institute  Evaluation of CQC’s national maternity inspection programme (final report, learning resource) | An evaluation of the of the Care Quality Commission’s (CQC) national maternity inspection programme | The objective was to characterise what good safety culture looks like. | Overlapping category papers | Maternity Services, NHS, England | A range of methods was used, including literature review, workshops with maternity service users, and a consultative survey, a previous framework on the features of safe maternity units was matured into a practical learning resource to support improvement in maternity care. | Core themes:  1. Commitment to safety and improvement at all levels, with everyone involved. 2. Technical competence that is attentive to diverse health needs and supported by formal training and informal learning  3. Optimised teamwork, team communication and coordination  4. Constant reinforcing of inclusive, respectful and ethical behaviours towards all colleagues and all maternity service users  5. Clear, respectful communication and shared decision-making with maternity service users that is culturally competent and accounts for diversity  6. Multiple problem-sensing systems, used as basis of action, and supported by active nurturing of the conditions for psychological safety  7. Structures, systems and processes designed for safety and regularly reviewed and optimised, guided by human factors / ergonomics principles where appropriate  8. High quality leadership and management |
| Todd, A. L., Ampt, A. J., & Roberts, C. L. (2017)  “Very Good” ratings in a survey of maternity care: kindness and understanding matter to Australian women | Primary mixed-methods research. | To identify the most significant factors associated with high ratings of care by women in the three maternity periods (antenatal, birth, and postnatal) | High rates of maternal wellbeing | New South Wales, Australia. Women who gave birth in seven different public hospitals. | A mixed-methods postal survey to 2048 women in NSW who had recently given birth. Questions included women’s overall ratings of their care, maternal characteristics and care factors. | Women's perceptions about the quality of their interpersonal interactions with health caregivers have a significant bearing on women's views about their maternity care journey. |
| Turner, S., Crowther, S., & Lau, A. (2022)  A grounded theory study on midwifery managers’ views and experiences of implementing and sustaining continuity of carer models within the UK maternity system | Primary qualitative research | To develop a theoretical framework that represents midwifery managers’ experiences of implementing and  sustaining MCoCer models within the UK’s National Health Service (NHS).” | Overlapping category papers | UK maternity system | Grounded theory (data was gathered by doing interviews) | Providing the appropriate support for MCoCer is time consuming and personally demanding for midwifery managers, however, implementing and sustaining MCoCer was shown by participants who valued MCoCer models to be rewarding, bringing meaning to their midwifery leadership.” |
| West, M., Bailey, S., & Williams, E. (2020)  The courage of compassion. Supporting Nurses and Midwives to Deliver High-Quality Care | Independent report commissioned by the RCN foundation (charity) | This review investigated how to transform nurses’ and midwives’ workplaces so that they can thrive and flourish and are better able to provide the compassionate, high-quality care that they wish to offer. | High rates of staff wellbeing | All nursing and midwifery staff and students across all four UK countries and all types of employers (e.g. NHS, GP practices, local authorities, independent sector), all areas of practice and care settings where nursing and midwifery staff operate (e.g. primary and secondary care, mental health, community care and social care. | A literature review, secondary analysis of quantitative data, semi-structured interviews and focus groups | - Nurses and midwives have three core work needs ensuring wellbeing and motivation at work, and minimising workplace stress: Autonomy, belonging, and contribution.  - 8 key recommendations influencing the workplace experience (1. Authority, empowerment, and influence 2. Justice and fairness 3. Work conditions and working schedules 4. Teamworking 5. Culture and leadership 6. Workload 7. Management and supervision. |
| West, M., & Coia, D. (2019).  Caring for doctors, caring for patients. | Independent report commissioned by the General Medical Council. (UK-wide review) | To identify the stressors which are negatively impacting on the health and  wellbeing of the 304,000 General Medical Council (GMC) registered doctors  and 41,000 medical students in the UK and to  show how to transform doctors’ workplaces so they thrive and flourish and are better able to provide the compassionate and high-quality care they and their patients wish them to deliver. | High rates of staff wellbeing | The review aimed to take account of the experience of all doctors and medical  students working and learning within the UK’s healthcare systems, in both primary  and secondary care. | This review involved extensive analysis of research literature and data; and engagement across all four countries of the UK with individuals and organisations.  Case studies are used in the report. | To ensure wellbeing and motivation at work, and to minimise workplace stress, people have 3 core needs (and all 3 must be met)   a. Autonomy/control b. Belonging c. Competence   8 vital recommendations are proposed in the report. |
