# Supplementary material for: Understanding the contexts and mechanisms that drive high-quality maternity care outcomes within organisations: generating 11 initial programme theories as phase 1 of a realist review
Source: BMJ Open. 2026 Aug 31;16(8):e119351. doi: 10.1136/bmjopen-2026-119351 (PMC13536141; doi:10.1136/bmjopen-2026-119351)
Supplement: Supplementary file 5 [file bmjopen-16-8-s006.docx]

Table 1IPTs with corresponding candidate paper citations that informed their development

| IPT conceptual label | IPT | References |
| --- | --- | --- |
| 1. A listening, hearing, learning and acting culture: responding to staff and service user insights | IF organisations embed and endorse a central and systematic approach (e.g. investment in the required time and resources, central data intelligence gathering and sharing) to a listening, hearing, learning and acting culture* inclusive of service users and staff alike THEN staff are more likely to implement a shared sense of ownership and responsibility for continual service improvements LEADING TO a responsive, transparent, constructive, supportive mechanism for quality improvement LEADING TO sustained high-quality care that is adapted to the population and individual needs in which staff and women feel respected and heard, particularly in case of adverse events.  *Meaning: to listen and act on what is heard, troubleshooting, pre-empting adverse events and disseminating lessons learned. Fostering robust accountability to the local community. | Russell, K. (2018)  Turner, S. (2022)  Downe et al., 2010  The Healthcare Improvement Studies Institute, 2024  Renfrew, M. (2024)  Liberati et al. (2019)  West, M., & Coia, D. (2019)  West, M., Bailey, S., & Williams, E. (2020)  North East and North Cumbria Local Maternity and Neonatal System (2023)  Todd, A. L., Ampt, A. J., & Roberts, C. L. (2017)  Crowe, C., & Manley, K. (2019)  Miller, S. et al. (2016) |
| 2.The organisation of maternity care is centred on women’s values and individualised/personalised needs by resourcing staff effectively | IF maternity services organise and invest in high-value care from the perspectives of those using the services*, where care is coordinated by a named professional who works within an aligned multi-disciplinary team and all staff have the appropriate support and resources** to provide relational care and maternal choice*** across the spectrum THEN service users are able to participate in informed-decision making in a meaningful way while staff feel safe and able to discuss and justify personalised care plans (e.g. care that is ‘out of guidance’) LEADING TO higher quality care for all women, seamless pathways, better outcomes and experiences for women and staff and a better use of resources.  * Evidence-based and personalised, high value maternity care across the continuum and across all settings (which are appropriately and equitably resourced, ringfenced, and accessible* to all women)  ** All maternity staff are enabled to develop meaningful and trusting relationships with women, provide accessible evidence-based information clearly and impartially, listen closely to questions, preferences and decisions communicated by maternity service users, and respond to them in a clear and impartial way while mediating full scope safety & this care is led and coordinated by a lead care provider.  *** maternal choice is in place, both for the support of physiological processes, and for interventions where they are needed or wanted. | Russell, K. (2018)  De Leo, A. (2021)  Turner, S. (2022)  Downe et al., 2010  The Healthcare Improvement Studies Institute, 2024  Renfrew, M. (2024)  Kroll-Desrosiers et al. (2016)  West, M., & Coia, D. (2019)  West, M., Bailey, S., & Williams, E. (2020)  North East and North Cumbria Local Maternity and Neonatal System (2023)  Todd, A. L., Ampt, A. J., & Roberts, C. L. (2017)  Crowe, C., & Manley, K. (2019)  Rönnerhag, M., Severinsson, E., et al. (2018)  De Jonge, A., Downe, S., et al. (2019)  Miller, S. et al. (2016) |
| 3. High-quality maternity adopts a broader definition of safety (beyond just physical safety) | IF maternity organisations, leadership and staff adopt a perspective of safety that includes both technical/clinical safety and psychological, emotional and cultural safety as defined by all stakeholders involved in maternity care which is embedded throughout all governance structures intelligent data gathering*, and resourcing, THEN all stakeholders adopt and champion a broad perspective on safety, creating a clinically, emotionally, culturally and psychologically safe environment for all LEADING TO all four outcome indicators.  *Triangulation of data gathering (outcomes and service user feedback (in the short and long-term), staffing levels, resources, time, flexibility, etc.) | Russell, K. (2018)  Downe et al., 2010  The Healthcare Improvement Studies Institute, 2024  Renfrew, M. (2024)  West, M., & Coia, D. (2019)  West, M., Bailey, S., & Williams, E. (2020)  Todd, A. L., Ampt, A. J., & Roberts, C. L. (2017)  Crowe, C., & Manley, K. (2019)  Rönnerhag, M., Severinsson, E., et al. (2018)  De Jonge, A., Downe, S., et al. (2019) |
| 4. Visible and supportive leadership enhancing the workplace and safety culture | IF knowledgeable and skilled maternity leaders manage hierarchies flexibly and strategically*, role model, while being highly visible on the shop floor, supporting and setting clear standards for what is considered appropriate actions and decisions (including safe escalation routes), explaining how and why, allowing colleagues to develop their skills through reflection-in-action*** THEN staff feel supported and enabled to work autonomously, to develop their skills within the full scope of their practice in united, passionate, coordinated and stable teams LEADING TO actions taken that are based on the requirements of the situation rather than on hierarchies protecting organisational interests, LEADING TO better outcomes and experiences for women and staff, and a better use of resources.  * Achieving a balance between supporting the learning and allowing staff to make autonomous decisions.  ** hierarchies can be mobilised when needed to clarify social norms and behavioural standards but can be muted where not required  *** aligned with psychological safety and attendance to the three core needs of autonomy, belonging and contribution | Russell, K. (2018)  Turner, S. (2022)  The Healthcare Improvement Studies Institute, 2024  Liberati et al. (2019)  West, M., & Coia, D. (2019)  West, M., Bailey, S., & Williams, E. (2020)  Crowe, C., & Manley, K. (2019) |
| 5. All maternity care systems, processes, procedures and care are grounded in evidence-based frameworks | IF the organisation employs ongoing ‘best practice’*evidence-based frameworks to support all systems and processes including governance** led by appropriately skilled professionals, THEN an evidence-informed inclusive, collaborative, and participative approach can flourish encompassing full-scope safety, effectiveness, and person-centredness LEADING TO the adoption, delivery and sustainability of evidence-based person-centred care in which providers can make the right decisions at the right time based on women’s choices and needs.  *‘Best Practice’: Evidence-based practice which exists of the triad of woman’s needs, best available clinical evidence and clinical expertise.  **e.g. for guideline development, the team should have appropriate research (systematic review) skills. (e.g. a fundamental valuing and reinforcement that all these things should be done by people with the appropriate knowledge and skills). | Russell, K. (2018).  De Leo, A. (2021).  McGowan et al., (2023)  The Healthcare Improvement Studies Institute, 2024  Renfrew, M. (2024)  De Jonge, A., Downe, S., et al. (2019)  Miller, S. et al. (2016 |
| 6. The system & workplace environment supports optimal clinical performance (operational fitness) | IF organisations design, implement and continually assess / review their working environments including systems, processes, structures, equipment, IT and facilities are optimised aligned with human factors/ergonomics* and Safety II principles and deploy intelligent data gathering (soft and hard) to manage system risk THEN the safety of the ‘system’ is enhanced/optimised with features of increased flexibility, preparedness and responsiveness to actual events/needs of the service (rather than reactive) LEADING TO increased staff wellbeing, decreased cognitive burdens and enabling staff to work to their full scope of practice (e.g., using clinical skills not administrator skills) LEADING TO operational ‘fitness’ within an organisation and optimal care delivery where staff are enabled to do the right thing consistently, better teamwork and job satisfaction, retention of the workforce and high-quality care provision.  * Psychological safety and attendance to the three core needs of autonomy, belonging and contribution. | De Leo, A. (2021)  Turner, S. (2022)  The Healthcare Improvement Studies Institute, 2024  Renfrew, M. (2024)  Liberati et al. (2019)  West, M., & Coia, D. (2019)  West, M., Bailey, S., & Williams, E. (2020)  North East and North Cumbria Local Maternity and Neonatal System (2023)  Miller, S. et al. (2016) |
| 7. The organisation and its systems are adapted to staff needs | IF maternity care organisations ensure minimum standards for basic facilities and staff support* for all aligned with psychological safety and attendance to the three core needs of autonomy, belonging and contribution THEN this improves working conditions, optimises health and wellbeing for staff (as they feel valued, supported and empowered), LEADING TO optimal care delivery where staff are enabled to do the right thing consistently, better teamwork and job satisfaction, retention of the workforce and high-quality care provision.  * Examples of basic facilities and staff support: places and time to rest and sleep, access to nutritious food and drinks, the tools needed for staff to do their job (e.g. parking, MDT training, (clinical) support, implement educational packages, respect, autonomy, cultural safety, consideration of work schedules based on realistic forecasting and demands that accounts for fatigue, supports safe shift swapping and enables breaks consistently and also providing easily accessible workplace tools to support clinical activity e.g., Wi-Fi, mobile desktops and evidence-based clinical resources, pathways and guidelines that help staff to do the right thing, a practical lay-out of the ward, etc.) | Russell, K. (2018)  Turner, S. (2022)  Downe et al., 2010  The Healthcare Improvement Studies Institute, 2024  Renfrew, M. (2024)  West, M., & Coia, D. (2019)  West, M., Bailey, S., & Williams, E. (2020)  North East and North Cumbria Local Maternity and Neonatal System (2023) |
| 8. Leadership drives a high-quality service by attending to the triad of safety, service user experience and clinical effectiveness | IF maternity leaders ensure that all aspects of the quality triad – safety, patient experience / person-centredness and clinical effectiveness are in place by authentically and proactively involving all staff and a wide range of service-users and acting on their views*; working with the local community and voluntary sector organisations THEN this facilitates strong, compassionate, and courageous leadership and management enabling the MDT to work well across the system LEADING TO a culture of caring, choice, and a strong service-user voice.  *e.g. intelligent data gathering and sharing is triangulated with all stakeholders to ensure accountability of senior teams | Russell, K. (2018)  McGowan et al., (2023)  The Healthcare Improvement Studies Institute, 2024  Renfrew, M. (2024)  Liberati et al. (2019)  Kroll-Desrosiers et al. (2016)  Todd, A. L., Ampt, A. J., & Roberts, C. L. (2017)  Crowe, C., & Manley, K. (2019)  Rönnerhag, M., Severinsson, E., et al. (2018)  De Jonge, A., Downe, S., et al. (2019) |
| 9. Responsiveness to ongoing workload and acuity assessments accounting for high-value care and the different workplace contexts | IF organisations implement and sustain a programme to review workload in their organisations that takes account of the specific context and work that is done in different areas of the service (with appropriate skill mix) and the nature of high-value care from the perspectives of those using the service, THEN the service can be adequately and appropriately resourced across the board, teams are protected to provide their services while also tackling excessive work demands on staff LEADING TO sustainability in delivering safe, high-value, high-quality care and meeting core staff needs so service users receive high-quality, safe, and compassionate care. | Russell, K. (2018)  Turner, S. (2022)  Downe et al., 2010  The Healthcare Improvement Studies Institute, 2024  Renfrew, M. (2024)  Liberati et al. (2019)  West, M., & Coia, D. (2019)  West, M., Bailey, S., & Williams, E. (2020)  Crowe, C., & Manley, K. (2019)  De Jonge, A., Downe, S., et al. (2019)  Miller, S. et al. (2016) |
| 10. Resource management for meaningful choice provision and optimal care with seamless care pathways for all service user needs | IF organisations implement and sustain evidence-based, responsive, and personalised, high value maternity care across the continuum and across all settings (which are appropriately and equitably resourced, ringfenced, and accessible* to all women) THEN seamless pathways exist for all women (e.g., both a ELCS AND homebirth pathway are available), meeting individual needs whilst addressing inequalities LEADING TO women and babies being offered the right care at the right time LEADING TO improved outcomes, improved wellbeing, optimal use of resources.  * Meaning: assurance of geographical, social, and economic access for all women. | Russell, K. (2018).  De Leo, A. (2021)  Turner, S. (2022)  McGowan et al., (2023)  The Healthcare Improvement Studies Institute, 2024  Renfrew, M. (2024)  Kroll-Desrosiers et al. (2016)  Crowe, C., & Manley, K. (2019)  Rönnerhag, M., Severinsson, E., et al. (2018)  De Jonge, A., Downe, S., et al. (2019)  Miller, S. et al. (2016) |
| 11. A culture of equitable care underpins all other IPTs | IF the maternity service engages in meaningful ways with marginalised and minoritised groups, including socially disadvantaged and the global majority and commits to co-producing anti-racist policies and practices* while engaging local voluntary groups and the local community sector and providing cultural safety training for staff THEN the culture of the organisation and frontline staff perpetuate inclusive, respectful and ethical behaviours LEADING TO improving the quality, safety and inclusiveness of services for these groups LEADING TO staff and service users from the Global Majority communities feeling and being safer and having better experiences, improved staff work experience.  *e.g. culturally appropriate interpretation and/or doula services ensuring continuity. | McGowan et al., (2023)  The Healthcare Improvement Studies Institute, 2024  Renfrew, M. (2024)  Kroll-Desrosiers et al. (2016) |
